# Supplementary material for: Space-confined synthesis of sinter-resistant high-entropy nanoparticle library
Source: Nat Commun. 2025 Aug 11;16:7383. doi: 10.1038/s41467-025-62729-3 (PMC12339747; doi:10.1038/s41467-025-62729-3)
Supplement: Supplementary file 1 — Supplementary Information [file 41467_2025_62729_MOESM1_ESM.pdf]

Supplementary Information

Space-confined synthesis of sinter-resistant high-entropy nanoparticle library

Shaoqing Chen[1,2], Xia Li[2]*, Ziqiang Qu[2], Xiang Li[3], Yuanzhu Gao[4], Peng-Fei Liu[5], Zhi-Qiang Dong[3], Peng Yu[3], Qiming Sun[2]*, Shixue Dou[6], Zhongfan Liu[7], and Jingyu Sun[1]*

[1]*College of Energy, Soochow Institute for Energy and Materials Innovations, Key Laboratory of Advanced Carbon Materials and Wearable Energy Technologies of Jiangsu Province, Soochow University, Suzhou 215006, P. R. China.*

[2]*Innovation Center for Chemical Science, College of Chemistry, Chemical Engineering and Materials Science, Jiangsu Key Laboratory of Advanced Negative Carbon Technologies, Soochow University, Suzhou 215123, P. R. China.*

[3]*Department of Mechanics and Aerospace Engineering, Southern University of Science and Technology, Shenzhen 518055, P. R. China.*

[4]*Cryo-electron Microscopy Center, Southern University of Science and Technology, Shenzhen, Guangdong, P. R. China.*

[5]*Institute of High Energy Physics, Chinese Academy of Sciences, Beijing 100049, China; Spallation Neutron Source Science Center, Dongguan 523808, P. R. China.*

[6]*Institute of Energy Materials Science, University of Shanghai for Science and Technology, Shanghai 200093, P. R. China.*

[7]*Center for Nanochemistry, College of Chemistry and Molecular Engineering, Peking University, Beijing 100871, P. R. China.*

*Corresponding author. E-mail address: sunjy86@suda.edu.cn; xiali@suda.edu.cn; sunqiming@suda.edu.cn

Supplementary Methods

As for aqueous precursor solutions, non-precious metal salts solutions (0.5 mol/L for each element) are mixed in equal volumes. For solutions containing precious metal, a small amount of H_2PtCl_6 or $PdCl_2$ solution is added. The mixed solution of Pt-quinary contains MCl_x (M is Mn, Fe, Co, Cu, and In) with an equal molar ratio and 1/12.5 molar ratio of H_2PtCl_6. The mixed solution of Pt-Senary contains MCl_x (M is Mn, Fe, Co, Ni, Cu, and Zn) with an equal molar ratio and 1/12.5 molar ratio of H_2PtCl_6. The mixed solution of Pt Septenary contains MCl_x (M is Mn, Fe, Co, Ni, Cu, and In), $Ce(NO_3)_3$ with an equal molar ratio and 1/5 molar ratio of H_2PtCl_6. The mixed solution of Pt Septenary contains MCl_x (M is Mn, Fe, Co, Ni, Cu, and In), $Ce(NO_3)_3$ with an equal molar ratio and 1/10 molar ratio of $PdCl_2$.

Typical synthesis: To prepare HEOs@MCM-41, 1 ml of mixed metal salt solutions are added dropwise to 1 g of MCM-41 and thoroughly mixed in the incipient wetness impregnation process. For the synthesis of HE-NPs@ molecular sieves (MS), 1 ml of mixed metal salt solutions are added dropwise to 3 g of ZSM-5, NaY, HY, TS-1, MCM-22, Beta and thoroughly mixed in the incipient wetness impregnation process. Then, the molecular sieves with mixed metal salt solutions adsorbed by capillary effect are dried and selectively retain the metal salt species in the pores. The metal-salts@MS precursors are calcinated in a furnace preheated to 900 °C for about 60 seconds, and then quickly taken out and quenched in ice water.

High-throughput synthesis: 50 mg of metal-salts@MS precursors are placed in a self-made quartz tube group and calcined in a high-temperature in a furnace preheated to 900 °C for about 60 seconds, and then quickly taken out and quenched in ice water.

Material characterization.

Wide-angle X-ray diffraction patterns were collected on a Bruker D8 with Cu Kα radiation (40 kV, 40 mA) at room temperature. The X-ray absorption data at the Pt L_3-edge of the samples were recorded at room temperature in the fluorescent mode with a silicon drift fluorescence detector at beam line BL14W1 of the Shanghai Synchrotron Radiation Facility (SSRF), China. The XAS was Small-angle X-ray diffraction patterns were performed at the 1W1A beam line of Beijing Synchrotron Radiation Facility (BSRF). Transmission electron microscopy (TEM), scanning transmission electron microscopy (STEM) images, electronic tomography and corresponding energy dispersive x-ray spectroscopy (EDS) mapping were obtained with a

Talos F200X instrument equipped with four EDS signal detectors. Atomic-resolution STEM images were carried out in an aberration-corrected Titan Themis G2 (FEI). The metal loading of the HE-NPs@MS and MPOs/MS was determined with Inductively Coupled Plasma-Optical Emission Spectrometer (ICP-OES) analyses carried out on an Agilent 5100 instrument. Nitrogen adsorption/desorption measurements were carried out on a Micromeritics 2020 analyzer at 77.35 K after the samples were degassed at 350 °C under vacuum. The temperature-programmed reduction of hydrogen (H_2-TPR) experiments were performed using a Micromeritics AutoChem II 2920 automated chemisorption analysis unit equipped with a thermal conductivity detector (TCD) under helium flow. Typically, 100 mg of the catalyst was pretreated with He (30 mL/min) for 1 hat 300 °C, followed by cooling down to 50 °C; then the sample was reduced in a flow of 10% H_2/He (50 mL/min) mixture from 50 to 900 °C with a heating rate of 10 °C/min.

Catalytic testing.

The PDH experiments were performed in a quartz tubular fixed-bed reactor with a 13 mm inner diameter at atmospheric pressure. Before the dehydrogenation reaction, 0.1 g catalyst mixed with 1 g quartz sand was reduced at 550 °C under a H_2 flow of 50 mL for 30 min, and then fed with propane. Typically, the reaction gas mixture contained 25 vol % propane and a balance of N_2 (C_3H_8/N_2 = 12.5/37.5 mL·min-1), which gave a WHSV of 13.5 h^{-1}. The reaction products were analyzed by an online gas chromatograph equipped with a flame ionization detector (FID). The C_3H_8 conversion, and selectivity of the C_3H_6 are defined based on the total number of carbon atoms balance method:

$$C_3H_8\ conversion\left(mol\%\right)=\frac{moles\ of\ C_3H_8\ \ reacted}{moles\ of\ C_3H_8\ \ fed}\times 100\% \quad \text{(S1)}$$

$$C_3H_6\ selectivity\left(mol\%\right)=\frac{moles\ of\ product\ C_3H_6\ \ formed}{moles\ of\ C_3H_8\ \ reacted}\times 100\% \quad \text{(S2)}$$

The turnover frequency (TOF) of propane and formation rate (FR) of propylene are defined using the equations

$$TOF_{propane}=\frac{F_{C_3H_8}\times 60\times X\times M_{Pt}}{24.4\times m_{cat.}\times w_{Pt}} \quad \text{(S3)}$$

$$FR_{propylene} = \frac{F_{C_3H_8} \times 60 \times X \times Y}{24.4 \times m_{cat.} \times w_{Pt}} \times 100 \quad \text{(S4)}$$

where $F_{C_3H_8}$, *X, and Y* are the flow rate, the conversion of C_3H_8 and selectivity of C_3H_6, respectively; $m_{cat.}$, M_{Pt}, and w_{Pt} are the weight of reaction catalysts, the weight of supported Pt in the catalyst, and the Pt atomic weight, respectively.

Surface energy calculation.

The surface Gibbs free energy of a droplet with radius R (G_R) is:

$$G_R = \gamma_{LV} A_{LV,R} + \gamma_{SL} A_{SL,R} - \gamma_{SV} A_{SV,R} \quad \text{(S5)}$$

where γ_{LV}, γ_{SL}, γ_{SV}, and $A_{LV,R}$, $A_{SL,R}$, $A_{SV,R}$, are the interfacial tension and area of liquid-vapor (*LV*), solid-liquid (*SL*), solid-vapor (*SV*), respectively.

According to the Young's equation, the relationship between interfacial tension is:

$$\gamma_{SV} = \gamma_{SL} + \gamma_{LV} \cos\theta \quad \text{(S6)}$$

where θ is the contact angle of solid-liquid, and $\theta > 90^\circ$ on a non-wetting surface.

The volume (*V*) of the droplet, the area of liquid-gas ($A_{LV,R}$) interface, and the area of solid-liquid interface ($A_{SL,R}$) are calculated using the equations (S7-9).

$$V = \frac{1}{3}\pi R^3[4-(1+\cos\theta)^2(2-\cos\theta)] \quad \text{(S7)}$$

$$A_{SL,R} = \pi R^2(1-\cos^2\theta) \quad \text{(S8)}$$

$$A_{LV,R} = 2\pi R^2(1-\cos\theta) \quad \text{(S9)}$$

So, the surface Gibbs free energy (G_R) of a droplet with radius R is:

$$\begin{aligned} G_R &= \gamma_{LV} A_{LV,R} + (\gamma_{SL} - \gamma_{SV}) A_{SL,R} \\ &= R^2[\pi(\gamma_{SL} - \gamma_{SV})(1-\cos^2\theta) + 2\pi\gamma_{LV}(1-\cos\theta)] \end{aligned} \quad \text{(S10)}$$

Due to the constant total amount of material in the system, the total surface Gibbs free energy (G_{Total}) of all droplets is:

$$G_{Total} = \frac{1}{V} G_R = \frac{1}{R}[12 - 3\times(1+\cos\theta)^2(2-\cos\theta)]\times$$
$$[(\gamma_{SL} - \gamma_{SV})(1-\cos^2\theta) + 2\pi\gamma_{LV}(1-\cos\theta)] \propto \frac{1}{R} \quad \text{(S11)}$$

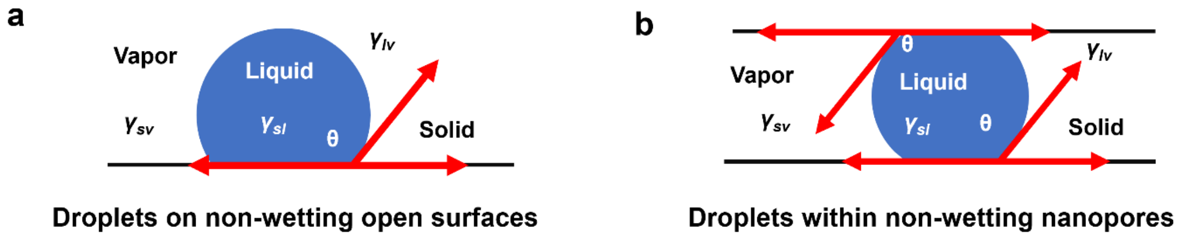

Supplementary Fig. 1: Interfacial tension of droplets (a) on non-wetting open surfaces and (b) within non-wetting nanopores.

When the droplets locate inside non-wetting nanopores, the expression of surface Gibbs free energy will change due to changes in the interfacial area and volume of the droplet. When the droplets are smaller than the nanopore, the morphology is similar to that in an open surface. So, the total surface Gibbs free energy (G_{Total}) of all droplets is still inversely proportional to the radius (R) of droplet.

When the size of the droplet is equivalent to the diameter of the nanopores (r), if the droplet continues to grow, it will form a geometric combination with two hemispheres at both ends and a cylinder in the middle because of the confinement effect of the nanopore.

The volume (V), the area of liquid-gas ($A_{LV,R}$) interface, and the area of solid-liquid interface ($A_{SL,R}$) of the droplet in the nanopores (r) with composite geometry are calculated using the equations (12-14). (S and C stand for the hemispheres and cylinder; the diameter of the nanopores is r; the height of the cylinder is h)

$$V = 2V_S + V_C$$
$$= \frac{2}{3}\pi(\frac{r}{2})^3(\frac{1}{1-\sin^2\theta})^{\frac{3}{2}} \times (1-\sin\theta)^2 \times (2+\sin\theta) + \pi(\frac{r}{2})^2 h \quad \text{(S12)}$$

$$A_{LV} = 2A_S = 4\pi(\frac{r}{2})^2 \times \frac{1}{1+\sin\theta} \quad \text{(S13)}$$

$$A_{SL} = 2\pi(\frac{r}{2}) \times h \quad \text{(S14)}$$

So, the surface Gibbs free energy (G) of composite geometry is:

$$
\begin{aligned}
G &= \gamma_{LV} A_{LV,R} + (\gamma_{SL} - \gamma_{SV}) A_{SL,R} \\
&= \gamma_{LV} \times 4\pi (\frac{r}{2})^2 \times \frac{1}{1+\sin\theta} + (\gamma_{SL} - \gamma_{SV}) \times 2\pi (\frac{r}{2}) h
\end{aligned}
\tag{S15}
$$

Due to the constant total amount of material in the system, the total surface Gibbs free energy (G_{Total}) of all droplets is:

$$
\begin{aligned}
G_{Total} &= \frac{1}{V} G = \frac{1}{\frac{2}{3}\pi (\frac{r}{2})^3 (\frac{1}{1-\sin^2\theta})^{\frac{3}{2}} \times (1-\sin\theta)^2 \times (2+\sin\theta) + \pi (\frac{r}{2})^2 h} \\
&\times [\gamma_{LV} \times 4\pi (\frac{r}{2})^2 \times \frac{1}{1+\sin\theta} + (\gamma_{SL} - \gamma_{SV}) \times 2\pi (\frac{r}{2}) h] \propto (m - \frac{1}{h})
\end{aligned}
\tag{S16}
$$

where m is a constant related to interfacial tension, contact angle, and pore diameter. So, the total surface energy (G_{Total}) is positively correlated with a constant minus the reciprocal of the height (h) of the middle cylinder.

Numerical Methods.

The fractional step lattice Boltzmann (FSLB) method, which originates from particle distribution functions and statistical physics principles, could simulate interfacial behavior through the evolution of equilibrium and non-equilibrium distribution functions. In this study, we employed FSLB to qualitatively simulate the thermodynamically driven spontaneous growth process and investigate the correlation patterns between final droplet size and diffusion kinetics, growth duration, and precursor concentration. It is worth noting that our use of the FSLB method is intended to capture the qualitative morphological evolution of droplets on open surfaces and within nanopores. The FSLB is limited in accurately describing heterogeneous systems and fluid behavior at the nanoscale. Therefore, our simulations are qualitative. To quantitatively assess the behavior of nanoalloys—where atomic-level segregation, surface energy differences, or element-specific interactions (e.g., oxygen affinity) may significantly affect physical behavior—atomistic modeling approaches such as molecular dynamics are required.

Governing equations for multiphase flows

To describe the flow property and the interface behavior of liquid metal droplet, the governing equations, i.e., Navier-Stokes equations and Cahn-Hilliard equation, are given as

$$
\frac{\partial \rho}{\partial t} + \nabla(\rho \boldsymbol{u}) \tag{S17}
$$

$$\frac{\partial \boldsymbol{u}}{\partial t}+\nabla\cdot(\boldsymbol{u}\boldsymbol{u})=-\frac{1}{\rho}\nabla p+\nabla[\nu\cdot(\nabla\boldsymbol{u}+(\nabla\boldsymbol{u})^T)]+\frac{1}{\rho}\boldsymbol{F}_s, \quad \text{(S18)}$$

$$\frac{\partial C}{\partial t}+\nabla\cdot(C\boldsymbol{u})=M\nabla^2\mu_C+Q_m, \quad \text{(S19)}$$

where ρ, $\mathbf{u}$, p and υ are the fluid density, velocity, pressure and kinematic viscosity, respectively. The surface force term $\mathbf{F}_s$ can be calculated by $\boldsymbol{F}_S=\mu_C\nabla C$, and C is the order parameter of Eq. (S15) which ranges from 0 to 1, μ_C is the chemical potential. The mass correction term Q_m imposed on the interface is used to maintain the mass conservation of the system [1, 2].

Original lattice Boltzmann equations

Generally, the multiphase lattice Boltzmann model with double distribution functions can be written as [3, 4]

$$f_\alpha(\boldsymbol{r}+\boldsymbol{e}_\alpha\delta t,t+\delta t)-f_\alpha(\boldsymbol{r},t)=-\frac{1}{\tau_f}\left(f_\alpha(\boldsymbol{r},t)-f_\alpha^{eq}(\boldsymbol{r},t)\right)+S_\alpha\delta t, \quad \text{(S20)}$$

$$g_\alpha(\boldsymbol{r}+\boldsymbol{e}_\alpha\delta t,t+\delta t)-g_\alpha(\boldsymbol{r},t)=-\frac{1}{\tau_g}\left(g_\alpha(\boldsymbol{r},t)-g_\alpha^{eq}(\boldsymbol{r},t)\right)+G_\alpha\delta t, \quad \text{(S21)}$$

with

$$f_\alpha^{eq}(\boldsymbol{r},t)=\rho\Gamma_\alpha(\boldsymbol{u})=\omega_\alpha\rho\left[1+\left(\frac{\boldsymbol{e}_\alpha\cdot\boldsymbol{u}}{c_s^2}+\frac{(\boldsymbol{e}_\alpha\cdot\boldsymbol{u})^2}{2c_s^4}-\frac{|\boldsymbol{u}|^2}{2c_s^2}\right)\right], \quad \text{(S22)}$$

$$g_\alpha^{eq}=\begin{cases}C-\frac{\mu_C M(1-\omega_0)}{c_s^2} & \alpha=0\\ \frac{\omega_\alpha(\mu_C M+C\boldsymbol{e}_\alpha\cdot\boldsymbol{u})}{c_s^2} & \alpha=1-8\end{cases}, \quad \text{(S23)}$$

$$S_\alpha=\left(1-\frac{1}{2\tau_f}\right)\frac{(\boldsymbol{e}_\alpha-\boldsymbol{u})}{c_s^2}\cdot[\Gamma_\alpha(\boldsymbol{u})\boldsymbol{F}_s-\nabla\rho c_s^2(\Gamma_\alpha(\boldsymbol{u})-\omega_\alpha)], \quad \text{(S24)}$$

$$G_\alpha=\left(1-\frac{1}{2\tau_g}\right)\omega_\alpha Q_m, \quad \text{(S25)}$$

$$\Gamma_\alpha(\boldsymbol{u})=\omega_\alpha\left[1+\frac{\boldsymbol{e}_\alpha\cdot\boldsymbol{u}}{c_s^2}+\frac{(\boldsymbol{e}_\alpha\cdot\boldsymbol{u})^2}{2c_s^4}-\frac{|\boldsymbol{u}|^2}{2c_s^2}\right], \quad \text{(S26)}$$

where $f_\alpha(\boldsymbol{r},t)$ and $g_\alpha(\boldsymbol{r},t)$ are the distribution functions of the flow field and the order parameter field with the lattice velocity $\mathbf{e}_\alpha$ and the weighting coefficient ω_α, $f_\alpha^{eq}(\boldsymbol{r},t)$ and $g_\alpha^{eq}(\boldsymbol{r},t)$ are the corresponding equilibrium distribution functions, δt denotes time interval, S_α and G_α represent the source terms for external force term and mass corrector, respectively, τ_f and τ_g are single relaxation parameters in the LB equation for the Navier-Stokes equations and the Cahn-Hilliard equation, respectively, which are related to the dynamic viscosity μ and the mobility M Through the Chapman-Enskog expansion analysis, the reconstructed governing

equations are given as follows:

$$\frac{\partial \rho}{\partial t}+\nabla\cdot\sum_\alpha \boldsymbol{e}_\alpha f_\alpha^{eq}=\boldsymbol{u}\cdot\nabla\rho, \tag{S27}$$

$$\frac{\partial \rho\boldsymbol{u}}{\partial t}+\nabla\cdot\sum_\alpha \boldsymbol{e}_{\alpha i}\boldsymbol{e}_{\alpha j}\left[f_\alpha^{eq}+\left(1-\frac{1}{2\tau_f}\right)f_\alpha^{neq}+\frac{\delta t}{2}S_\alpha\right]=\boldsymbol{F}_s, \tag{S28}$$

$$\frac{\partial C}{\partial t}+\nabla\cdot\sum_\alpha \boldsymbol{e}_\alpha g_\alpha^{eq}+\left(1-\frac{1}{2\tau_g}\right)\nabla\cdot\sum_\alpha \boldsymbol{e}_\alpha g_\alpha^{neq}=Q, \tag{S29}$$

where the non-equilibrium distribution functions are approximated as

$$f_\alpha^{neq}=f_\alpha-f_\alpha^{eq}\sim\varepsilon f_\alpha^{(1)}=-\tau_f\delta t\left[\left(\frac{\partial}{\partial t}+\boldsymbol{e}_\alpha\cdot\nabla\right)f_\alpha^{eq}-S_\alpha\right], \tag{S30}$$

$$g_\alpha^{neq}=g_\alpha-g_\alpha^{eq}\sim\varepsilon g_\alpha^{(1)}=-\tau_g\delta t\left[\left(\frac{\partial}{\partial t}+\boldsymbol{e}_\alpha\cdot\nabla\right)g_\alpha^{eq}-G_\alpha\right]. \tag{S31}$$

The detailed derivation of the Chapman-Enskog expansion analysis can be found in the previous work [5, 6].

Fractional step lattice Boltzmann equations

The fundamental concept of the fractional step lattice Boltzmann model involves the prediction of intermediate macroscopic physical variables through the moments of equilibrium distribution functions, followed by correction using the moments of non-equilibrium distribution functions and source terms, and the computational procedure can be described as follows [5, 6]:

(1) the prediction step:

$$\tilde{\rho}=\sum_\alpha f_\alpha^{eq}(\boldsymbol{r}-\boldsymbol{e}_\alpha\delta t,t-\delta t), \tag{S32}$$

$$\tilde{\rho}\tilde{\boldsymbol{u}}=\sum_\alpha \boldsymbol{e}_\alpha f_\alpha^{eq}(\boldsymbol{r}-\boldsymbol{e}_\alpha\delta t,t-\delta t), \tag{S33}$$

$$\tilde{C}=\sum_\alpha g_\alpha^{eq}(\boldsymbol{r}-\boldsymbol{e}_\alpha\delta t,t-\delta t), \tag{S34}$$

(2) the correction step

$$\rho(\boldsymbol{r},t)=\tilde{\rho}-2\sum_\alpha f_\alpha^{neq}(\boldsymbol{r}-\boldsymbol{e}_\alpha\delta t,t), \tag{S35}$$

$$\rho(\boldsymbol{r},t)\boldsymbol{u}(\boldsymbol{r},t)=\tilde{\rho}\tilde{\boldsymbol{u}}-\sum_\alpha \boldsymbol{e}_\alpha\left[\left(1-\frac{1}{\tau_f}\right)f_\alpha^{neq}(\boldsymbol{r}-\boldsymbol{e}_\alpha\delta t,t)+\delta t S_\alpha\right]+\boldsymbol{F}_s\delta t, \tag{S36}$$

$$C=\tilde{C}-\left(1-\frac{1}{\tau_g}\right)\sum_\alpha g_\alpha^{neq}(\boldsymbol{r}-\boldsymbol{e}_\alpha\delta t,t)+Q\delta t. \tag{S37}$$

Through the Taylor series expansion of above equations, the reconstructed governing equations can be recovered within a second order accuracy, the relevant derivation can be referred in previous work [5, 6].

The physical condition

The density ratio and the viscosity ratio used in this simulation are equal to 5000 and 100, respectively. The surface tension coefficient is 0.53 N/m. The interfacial thickness and the

mobility coefficient are set as 4 and 0.1, respectively. The computational domain is discretized by 800×140 lattice units, and the no-slip boundary condition is applied on the top and bottom boundaries, while the periodic boundary condition is imposed on the left and right boundaries. This grid size is sufficiently small to produce a grid-independent solution. Besides, to consider the wettability effect of the channel, a geometric method is implemented on the top and bottom boundaries in present simulation, and the contact angle of two-dimensional droplet can be given as

$$tan\left(\frac{\pi}{2}-\theta\right)=\frac{-\boldsymbol{n}\cdot\nabla C}{|\nabla C-(\boldsymbol{n}\cdot\nabla C)\boldsymbol{n}|}, \quad \text{(S38)}$$

Computational methods for the formation energy of oxygen vacancy.

First-principles calculations were performed within the framework of the Perdew-Burke-Ernzerhof generalized gradient approximation and projected-augmented wave approach, as implemented in the Vienna *ab initio* simulation package (VASP)[7-10]. The periodic solution of the crystal was represented by using Bloch states with a Γ-centered k-spacing of 0.2 $Å^{-1}$ and 500 eV energy cutoff to give a force convergence of less than 0.01 eV/Å and energy less than 0.1 meV[11]. To simulate the alloying system, we employed the crystal cell with the stoichiometric ratio of 1:1:1:1:1:1:8 for Mn, Ni, Zn, Co, Fe, Cu, and O atoms, respectively, in which Mn, Ni, Zn, Co, Fe and Cu atoms dispersedly occupancy the Cu and Mn sites in the crystal of $CuMn_2O_4$. The energy of oxygen vacancy formation (E_{Vo}) was calculated according to the following formula:

$$E_{form} = E_{defect} - E_{host} + 0.5\times E_{O2} \quad \text{(S39)}$$

where E_{defect}, E_{host}, and E_{O2} are the total energy of the defect system, host system and oxygen, respectively. In the calculation, the sizes of the supercells are 4×4×2, 2×2×2, 2×2×1, 1×1×1, and 1×1×1 for ZnO, NiO, Fe_2O_3, Co_3O_4, and $MnFeCoNiCuZnO_8$, respectively.

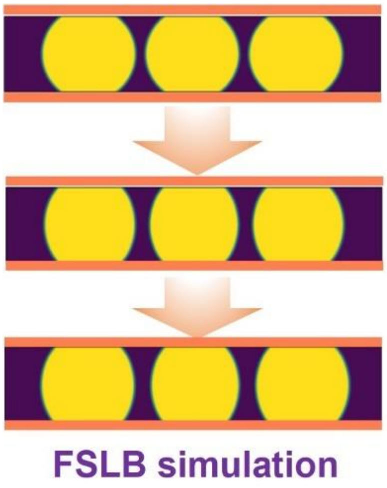

Supplementary Fig. 2: The simulation of the interface behavior of liquid metal droplet within non-wetting pores by a fractional step lattice Boltzmann (FSLB) method.

When multiple droplets have the same size equivalent to the pore, The FSLB simulation suggests that the droplets tend to remain independently.

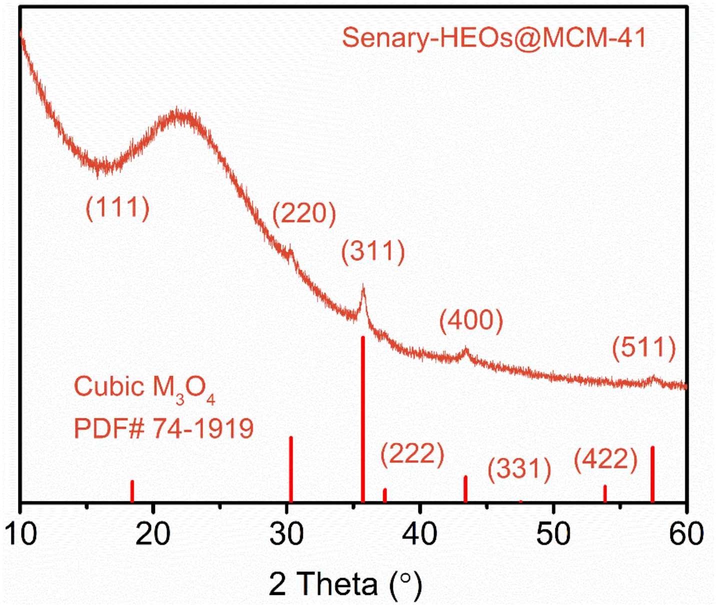

Supplementary Fig. 3: PXRD pattern of Senary-HEOs@MCM-41.

Notes: The PXRD pattern of Senary-HEOs@MCM-41 materials prepared via the ICQ process, which can be attributed to the cubic M_3O_4@MCM-41 (containing Mn, Fe, Co, Ni, Cu, Zn).

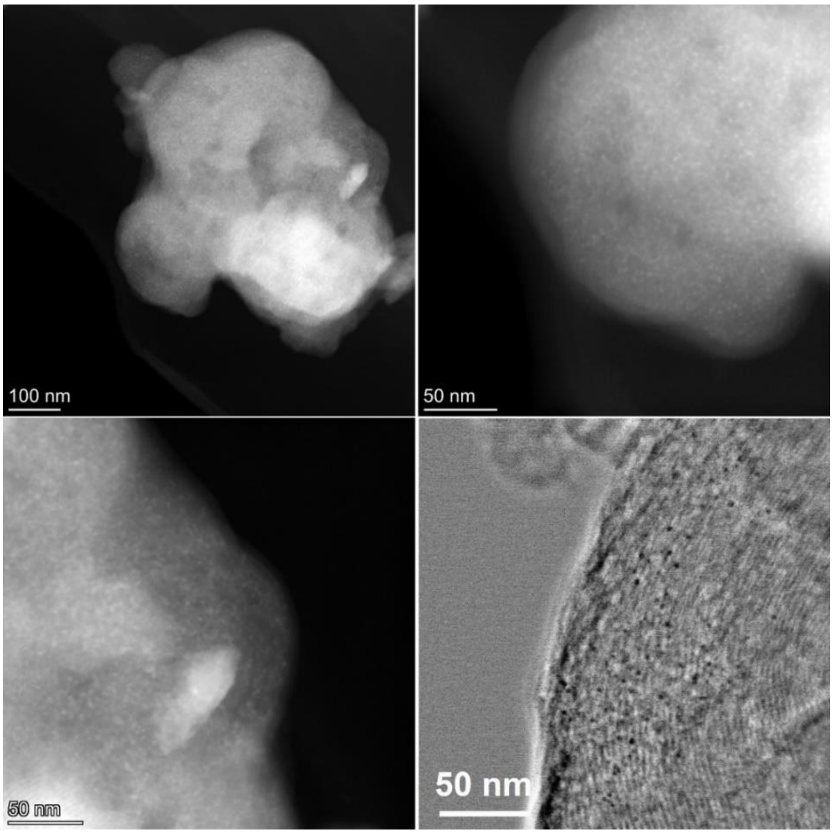

Supplementary Fig. 4: HAADF-STEM images of Pt-Quinary-HEOs@MCM-41.

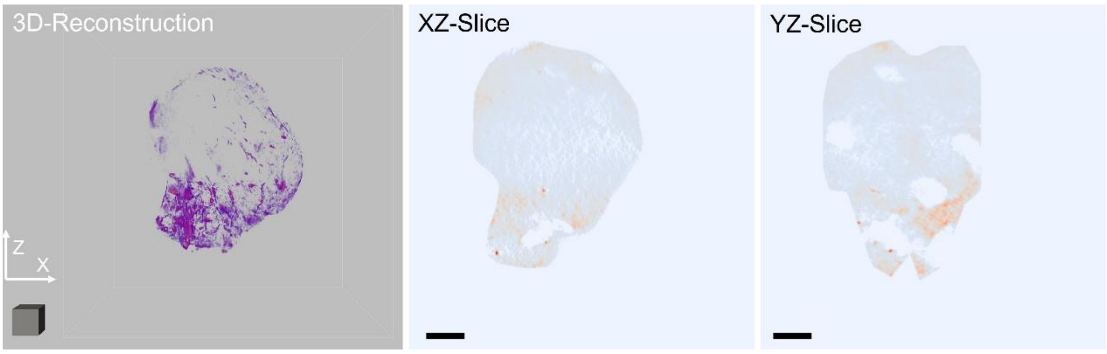

Supplementary Fig. 5: Electron tomography of Pt-quinary-HEOs@MCM-41. The color map of segmented reconstruction and representative tomograms (The red dots represent metal particles). Scale cube, 50^3 nm^3; Scale bar, 50 nm.

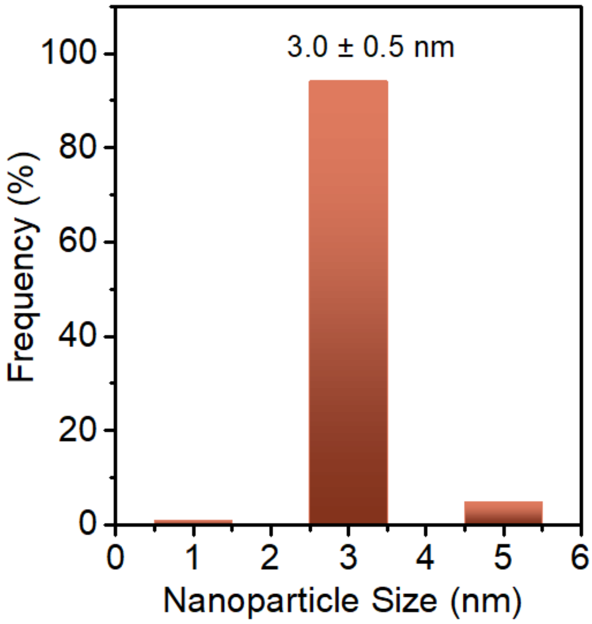

Supplementary Fig. 6: Size distributions of HEO nanoparticles confined in MCM-41 of Pt-Quinary-HEOs@MCM-41.

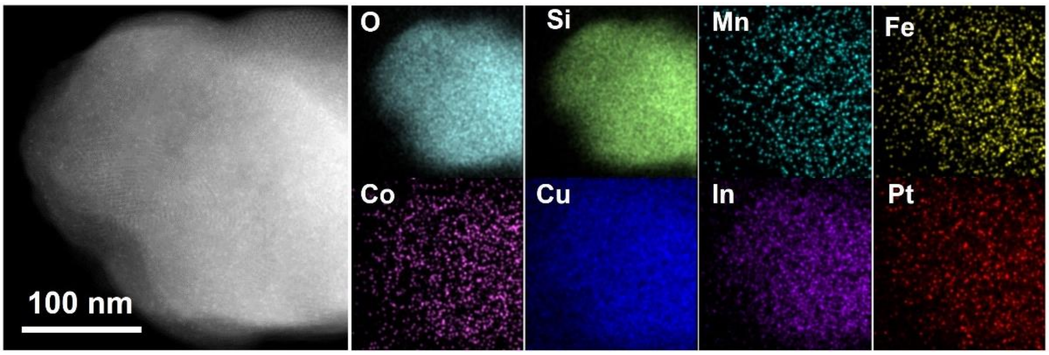

Supplementary Fig. 7: HAADF-STEM image and elemental maps of Pt-Quinary-HEOs@MCM-41.

Notes: The results demonstrate that no large metal particles are found on the surface, and the elements of Mn, Fe, Co, Cu, In and Pt are distributed homogeneously, indicating the establishment of HE-NP system.

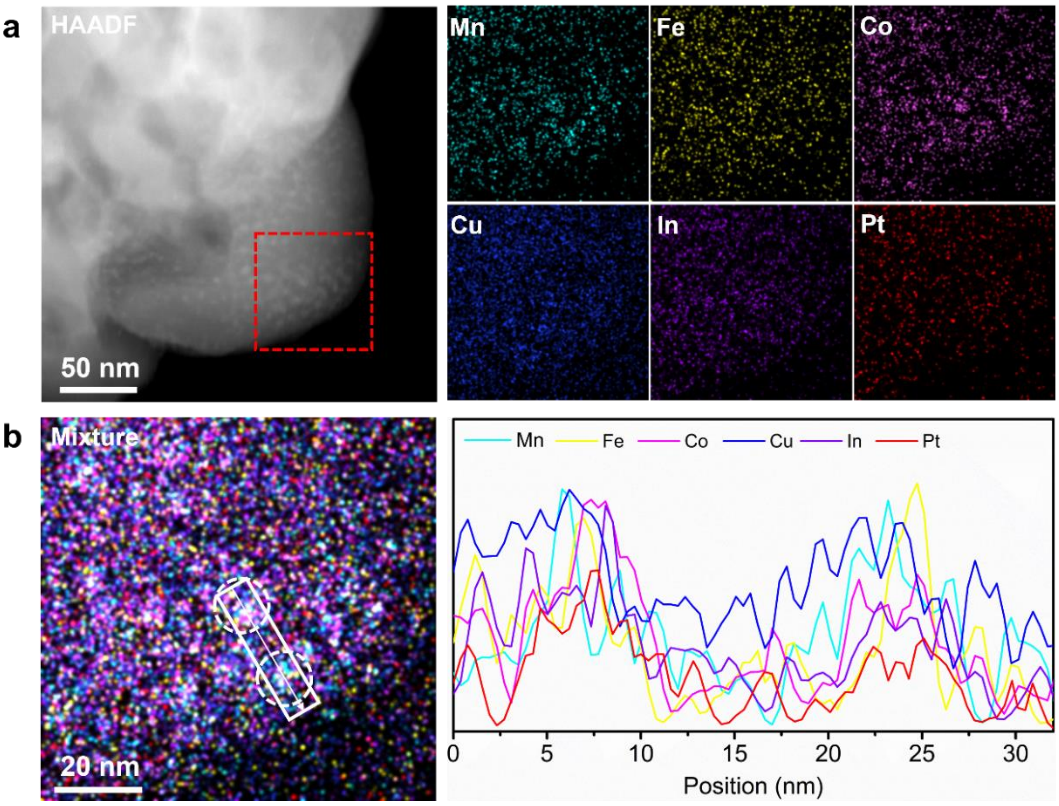

Supplementary Fig. 8: (a) Elemental maps and (b) corresponding line scan profiles for nanoparticles of Pt-Quinary-HEOs@MCM-41.

Notes: The elemental mapping and line scan profiles of Pt-Quinary-HEOs@MCM-41demonstrate that multiple metal elements are homogeneously distributed within individual particles.

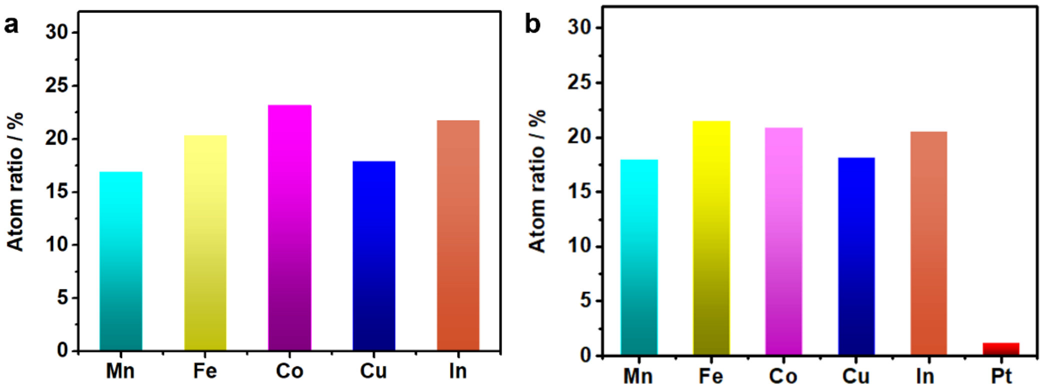

Supplementary Fig. 9: Metallic compositions of (a) Quinary-HEOs@MCM-41 and (b) Pt-Quinary-HEOs@MCM-41.

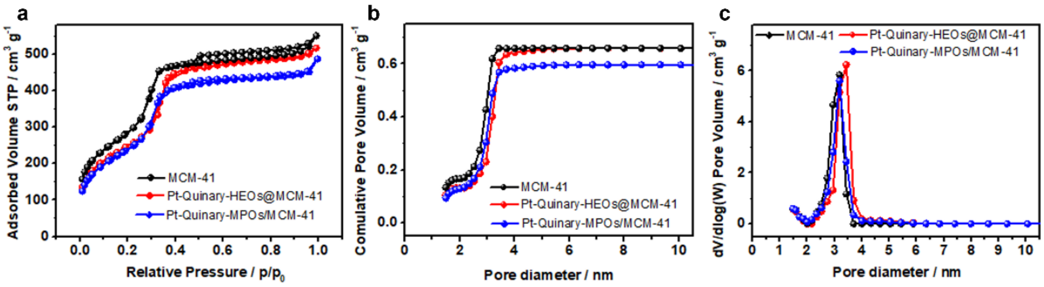

Supplementary Fig. 10: (a) N_2 adsorption/desorption isotherms, (b) total pore capacity, and (c) pore size distributions of MCM-41, Pt-Quinary-HEOs@MCM-41 and Pt-Quinary-MPOs/MCM-41.

Notes: The analysis of the pore structure shows that the pore structure still maintains a regular mesoporous structure after loading metal oxides. The total pore capacity of Pt-Quinary-HEOs@MCM-41 is equivalent to MCM-41, while the total pore capacity of and Pt-Quinary-MPOs/MCM-41 has slightly decreased (Table S1). The slight increase in pore size of Pt-Quinary-HEOs@MCM-41 may be attribute to the slight erosion of the pore wall by liquid metal inside the pores during the formation of HE-NP particles.

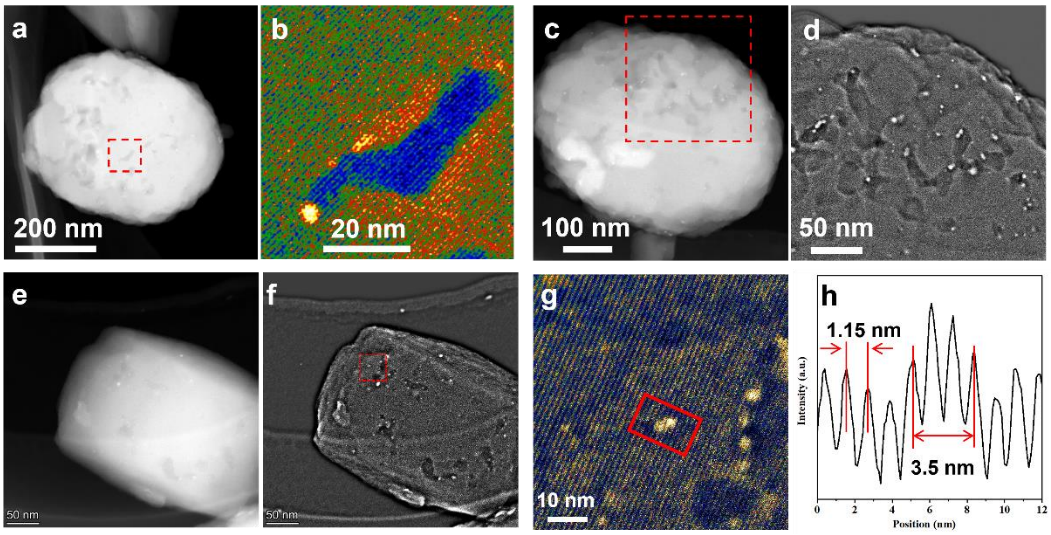

Supplementary Fig. 11: (a, c, d, e, f) HAADF-STEM images and (b, g) high-resolution STEM images of Pt-Senary-HEOs@ZSM-5; (h) Intensity profile corresponding to the red-boxed region in g.

Notes: The STEM images show the morphology of Pt-Senary HEOs@ZSM-5, in which the uniform nanoparticles are confined in ZSM-5 with partial destruction of frameworks. The STEM images demonstrate that some of the microporous walls are broken and irregular grooves resembling earthworm holes are formed, which can be attributed to the flow erosion of liquid metal. The “earthworm-holes” provide spaces for HEO nanoparticles which are slightly larger than the size of the cages of microporous MS, while most of the pores of the MS are still intact with a clear lattice of pore wall.

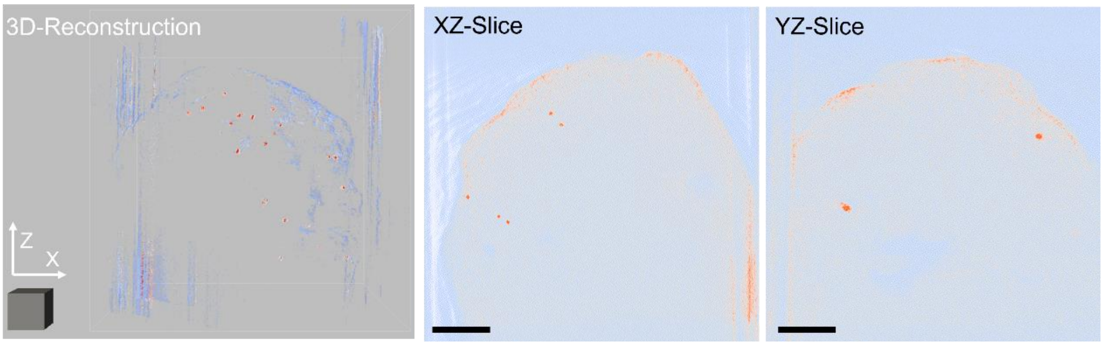

Supplementary Fig. 12: Electron tomography of Senary-HEOs@ZSM-5. The color map of segmented reconstruction and representative tomograms (The red dots represent metal particles). Scale cube, 50^3 nm^3; Scale bar, 50 nm.

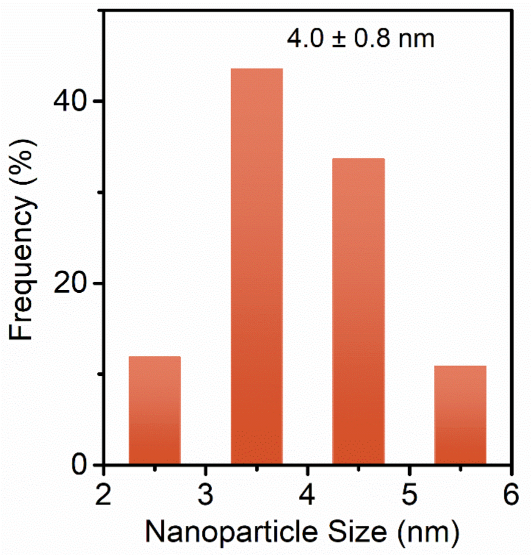

Supplementary Fig. 13: Size distributions of HEO nanoparticles confined in ZSM-5 of Pt-Senary-HEOs@ZSM-5.

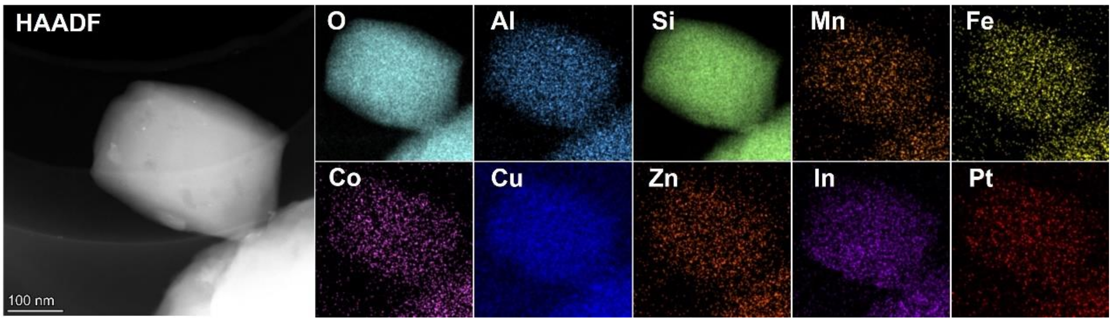

Supplementary Fig. 14: HAADF-STEM image and elemental maps of Pt-Senary-HEOs@ZSM-5.

Notes: No large metal particles are found on the surface, and the elements of Pt, Mn, Fe, Co, Cu, Zn, and In are distributed homogeneously throughout the MS, indicating the establishment of a HE-NPs system.

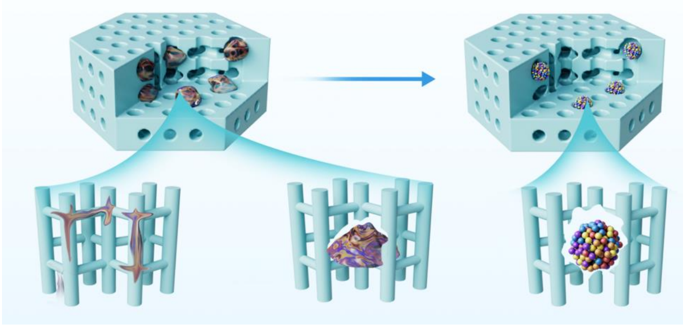

Supplementary Fig. 15: Schematic illustrating the formation of HEOs within micropores of MSs.

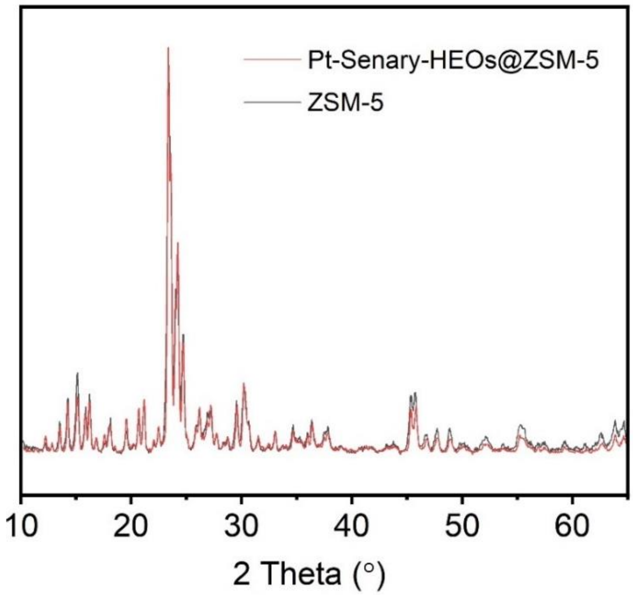

Supplementary Fig. 16: PXRD pattern of Pt-Senary-HEOs@ZSM-5 sample prepared by ICQ strategy.

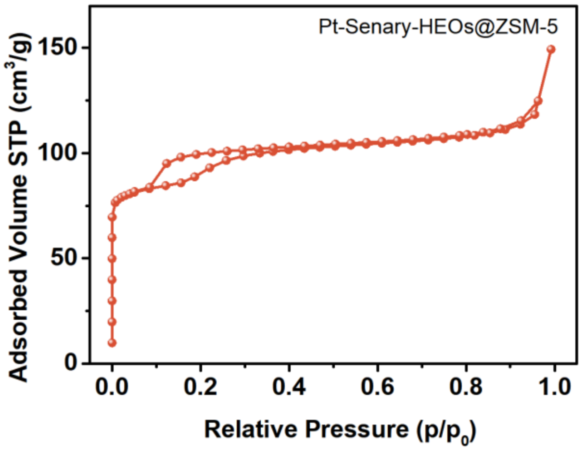

Supplementary Fig. 17: N_2 adsorption/desorption isotherms of Pt-Senary-HEOs@ZSM-5.

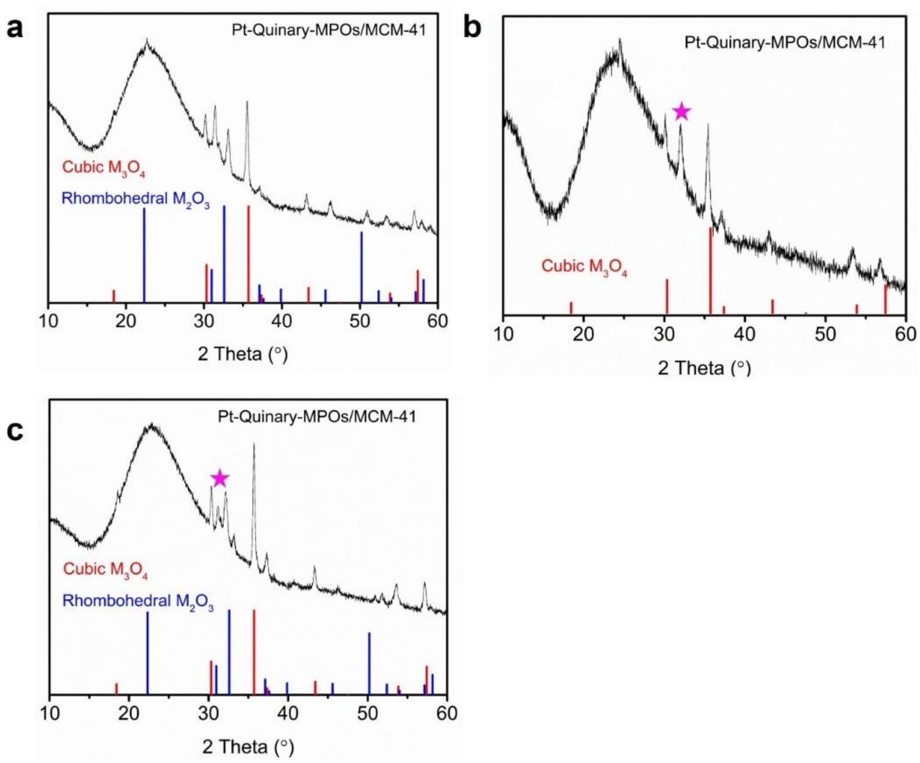

Supplementary Fig. 18: PXRD patterns of Pt-Quinary-MPOs/MCM-41 samples prepared by (a) traditional impregnation, calcination, and anneal process. (b) traditional impregnation, calcination, and quench process. (c) incipient wetness impregnation, calcination, and anneal process.

Notes: As for the Pt-Quinary-MPOs/MCM-41 samples prepared by traditional impregnation, calcination, anneal or quench process, the peaks of XRD pattern are attributed to cubic M_3O_4 (PDF# 74-1919), rhombohedral M_2O_3 (PDF# 73-1809) and other impurity oxides (⋆). For the Pt-Quinary-MPOs/MCM-41 sample prepared by incipient wetness impregnation, calcination, and anneal process, the peaks of XRD pattern are attributed to cubic M_3O_4, rhombohedral M_2O_3 and other impurity oxides (⋆). The sharp peaks indicate the generation of large particles, which is consistent with TEM results.

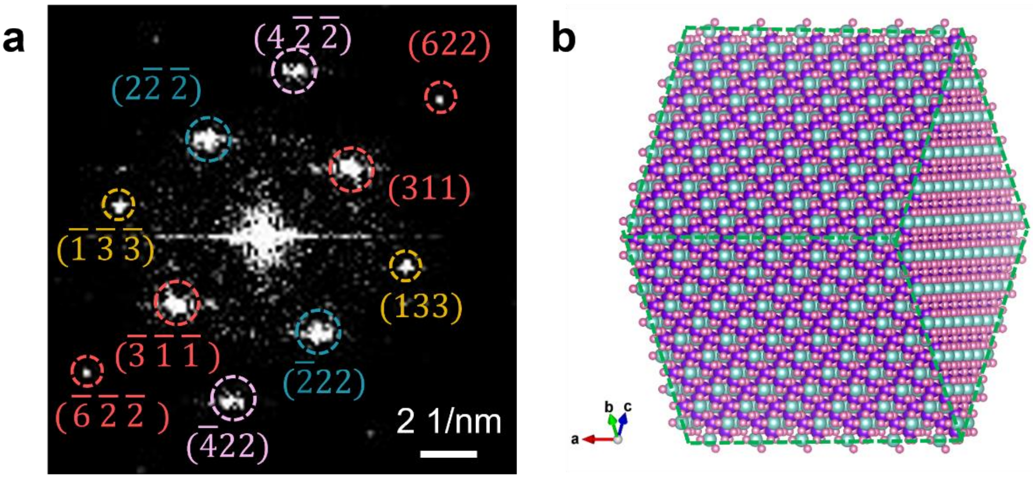

Supplementary Fig. 19: (a) Fast Fourier transform (FFT) analysis of the HEO nanoparticle in Fig. 2f, and (b) the model of spinel structured cubic phase M_3O_4.

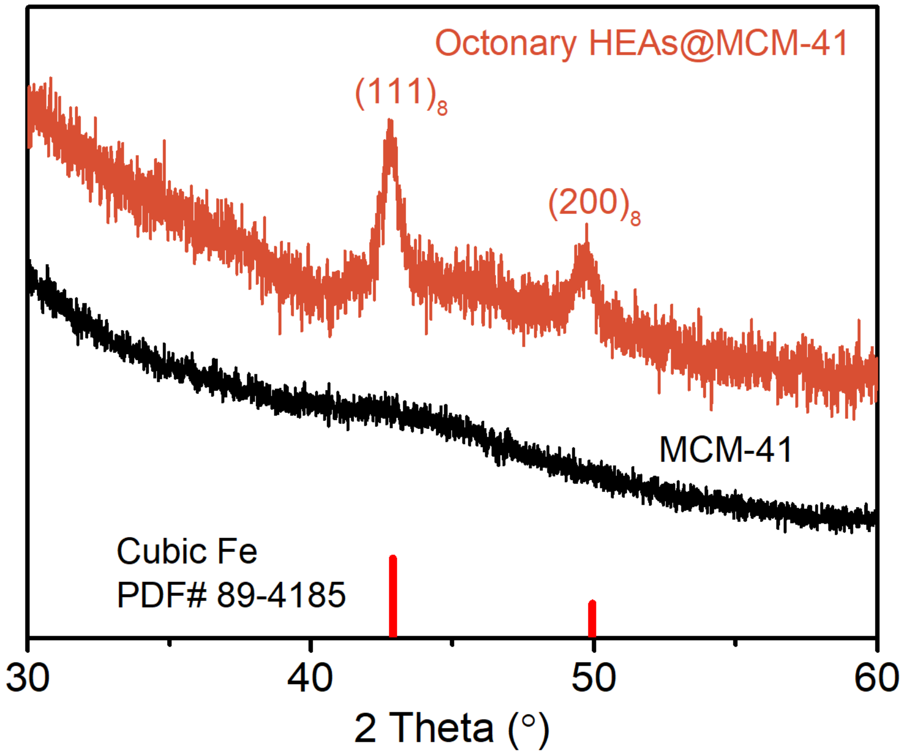

Supplementary Fig. 20: PXRD pattern of Octonary-HEAs@MCM-41 prepared by ICQ strategy with sealing the precursor inside a vacuum quartz tube.

Notes: When the precursor powder is sealed in a vacuum quartz tube, the obtained sample by ICQ is a single-phase cubic structure HEA, as shown in the XRD pattern. This result suggests that when preparing samples through the ICQ strategy, high entropy materials are mainly oxidized by oxygen in the air.

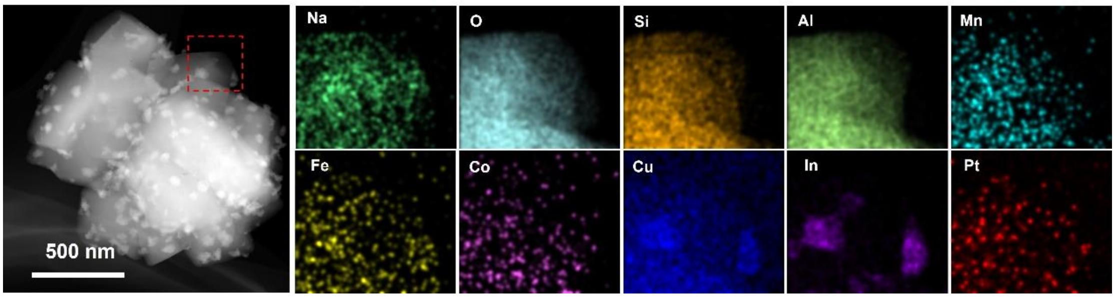

Supplementary Fig. 21: HAADF-STEM image and elemental maps of Pt-Quinary-MPOs/NaY.

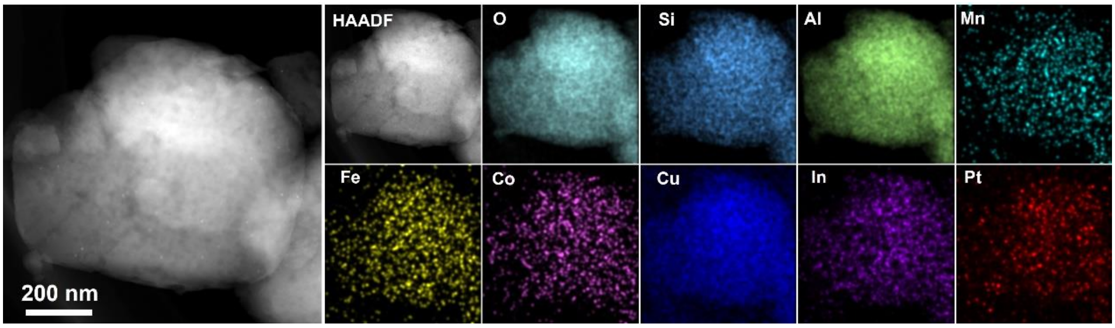

Supplementary Fig. 22: HAADF-STEM image and elemental maps of Pt-Quinary-HEOs@HY.

Notes: No large metal particles are found on the surface of HY, and the elements of Mn, Fe, Co, Cu, In and Pt are distributed homogeneously throughout the HY, which is different with MPOs loading on NaY.

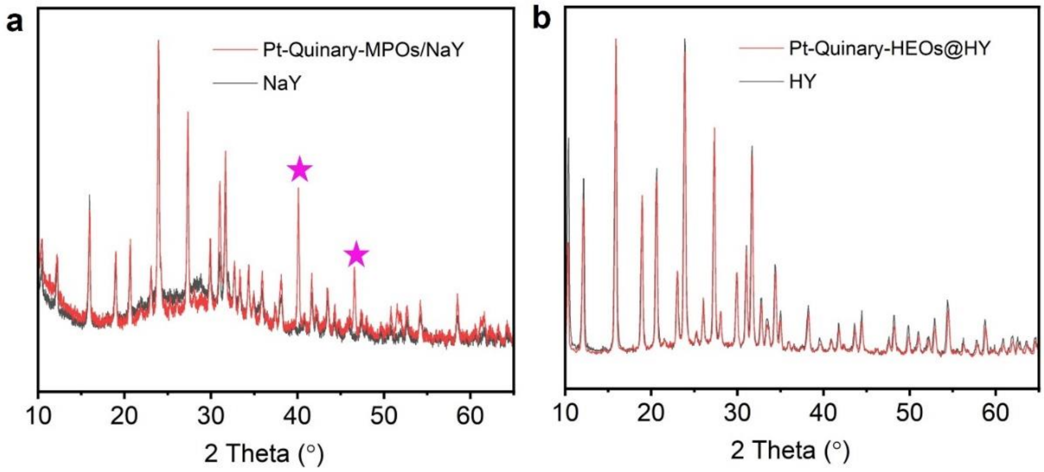

Supplementary Fig. 23: PXRD patterns of (a) Pt-Quinary-MPOs/NaY and (b) Pt-Quinary-HEOs@HY samples prepared by ICQ strategy.

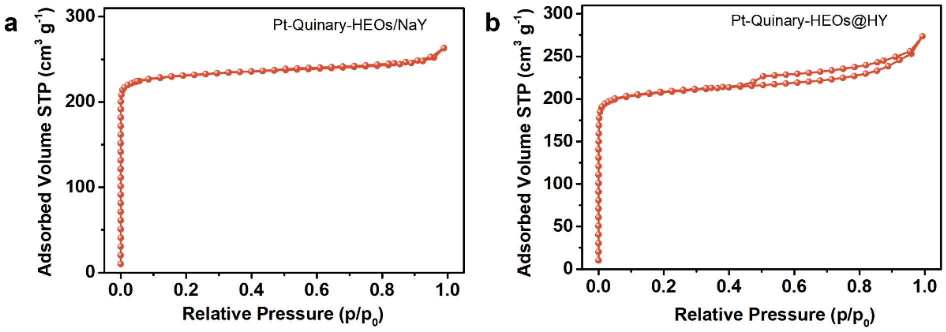

Supplementary Fig. 24: N_2 adsorption/desorption isotherms of (a) Pt-Quinary-HEOs/NaY and (b) Pt-Quinary-HEOs@HY.

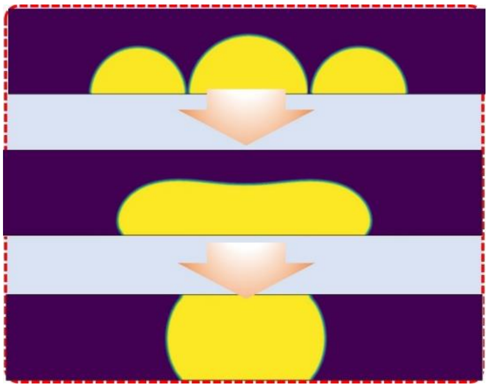

Supplementary Fig. 25: FSLB simulations of the high-loading liquid metal droplets within non-wetting nanopores.

Notes: The computational fluid dynamics simulations with FSLB method indicate that the liquid metal droplets in non-wetting nanopores could develop into one-dimensional nano-columns by augmenting the precursor loading.

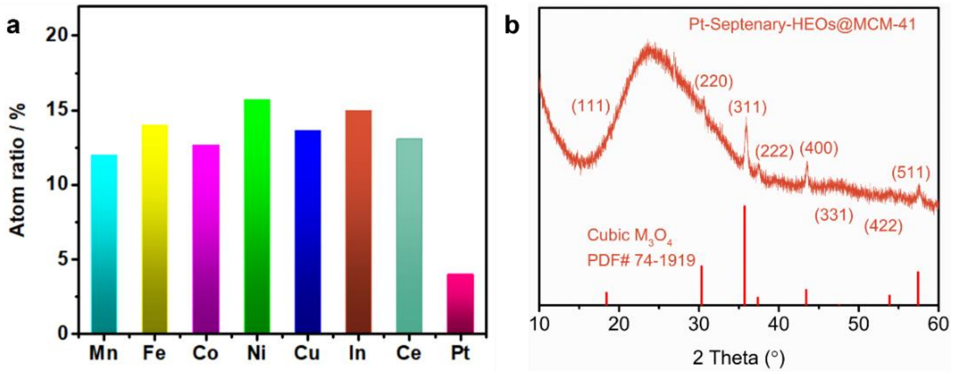

Supplementary Fig. 26: (a) Metallic compositions and (b) XRD pattern of Pt-Septenary-HEO@MCM-41 sample prepared by ICQ strategy (Metal content: 1.7 wt%).

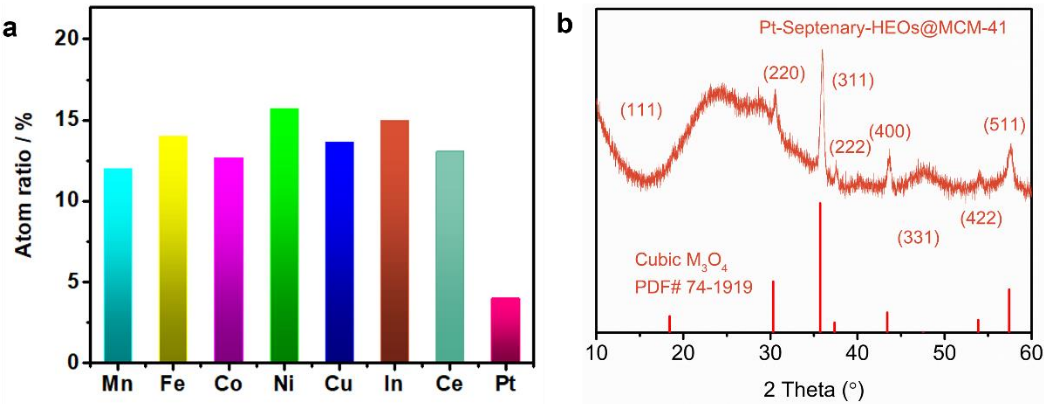

Supplementary Fig. 27: (a) Metallic compositions and (b) XRD pattern of Pt-Septenary-HEO@MCM-41 sample prepared by ICQ strategy (Metal content: 3.5 wt%).

Notes: When the metal loading increases, the aggregated metal droplets form a liquid column, leading to the formation of HEOs nanorods in the one-dimensional nanopores of molecular sieve after the quenching process. The XRD peak shows that the main structure of the metal species is still a spinel structured cubic phase HEO. The peak intensity increased, indicating an increase in metal loading and particle size, which is consistent with the TEM results. Meanwhile, weak CeO_2 broad peaks appeared in XRD, indicating that phase separation may occur in the sample when the impregnation amount exceeds the pore volume.

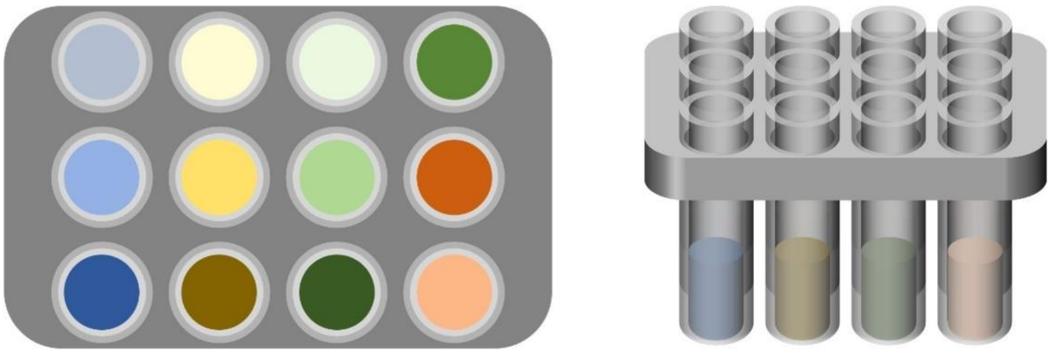

Supplementary Fig. 28: Schematic diagram of self-made high-throughput synthesis equipment.

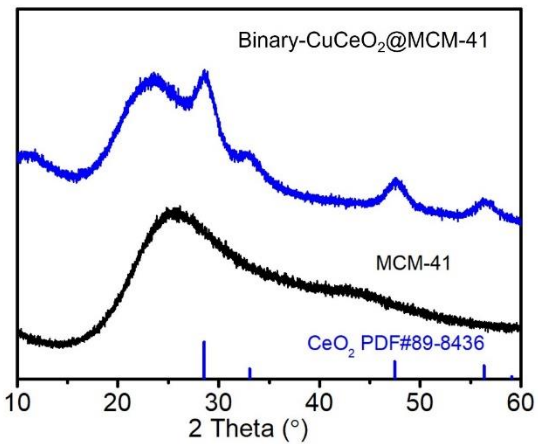

Supplementary Fig. 29: PXRD pattern of Binary-$CuCeO_2$@MCM-41 prepared by ICQ strategy.

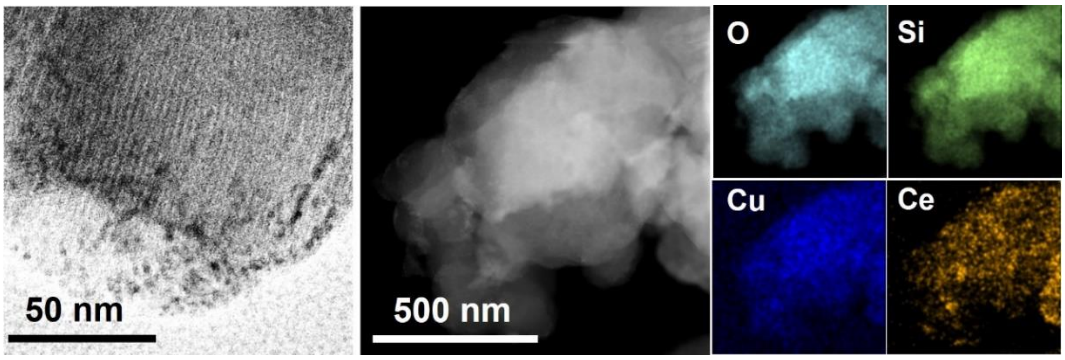

Supplementary Fig. 30: TEM, HAADF-STEM image and elemental maps of Binary-$CuCeO_2$@MCM-41.

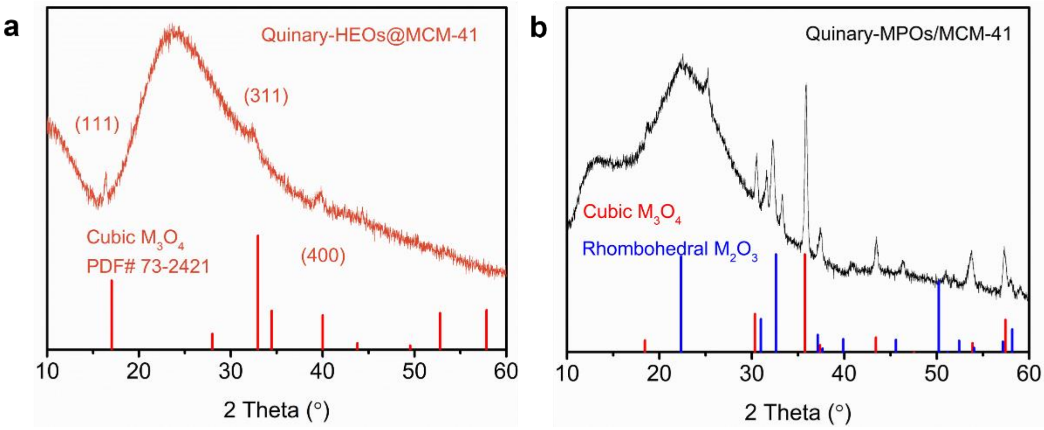

Supplementary Fig. 31: (a) The PXRD pattern of Quinary-HEOs@MCM-41 prepared by ICQ strategy. (b) PXRD pattern of Quinary-MPOs/MCM-41 prepared by incipient wetness impregnation, calcination, and anneal process.

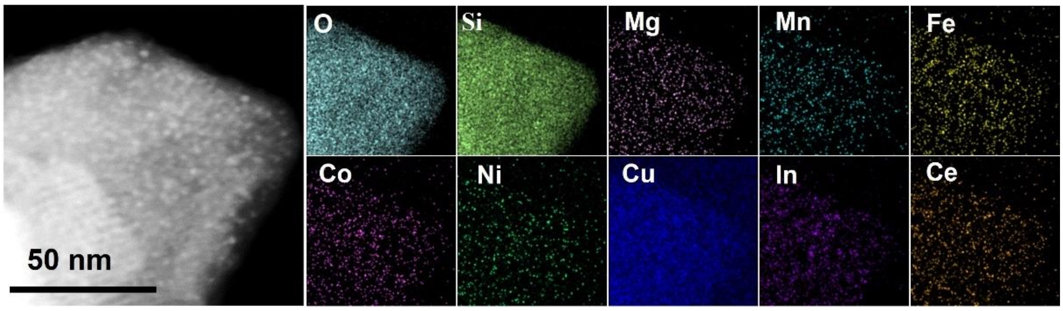

Supplementary Fig. 32: HAADF-STEM image and elemental maps of Octonary-HEOs@MCM-41.

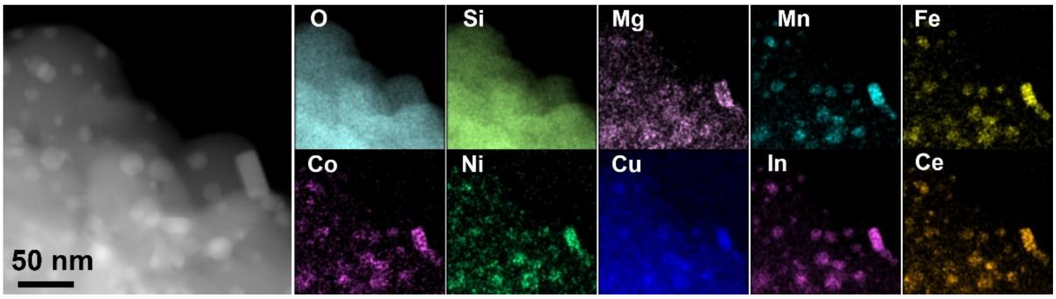

Supplementary Fig. 33: HAADF-STEM image and elemental maps of Octonary-MPOs/MCM-41.

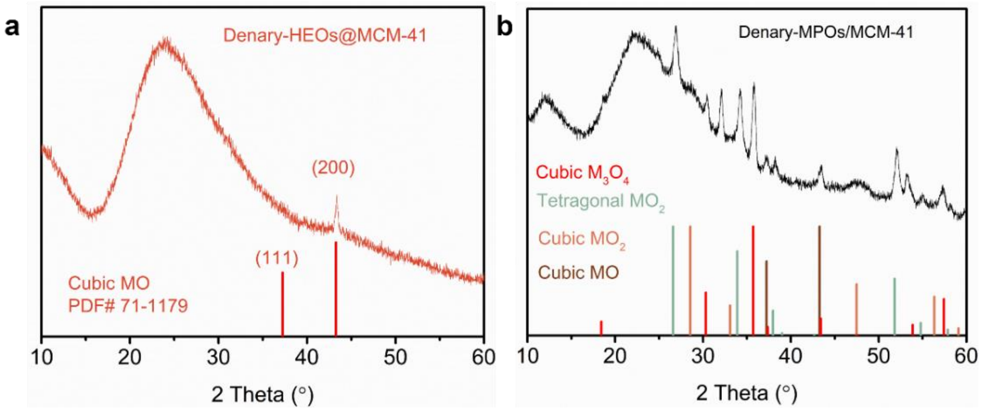

Supplementary Fig. 34: (a) The PXRD pattern of Denary-HEOs@MCM-41 prepared by ICQ strategy. (b) XRD pattern of Denary-MPOs/MCM-41 sample incipient wetness impregnation, calcination, and anneal process.

Notes: The XRD pattern of Denary-HEOs@MCM-41, containing 10 metal elements of Mn, Fe, Co, Ni, Cu, Zn, In, Sn, Sr, Ce, is attributed to the cubic phase MO, such as NiO (PDF# 71-1179), $Ni_{0.9}Cu_{0.1}O$ (PDF# 78-0645) and $Ni_{0.6}Zn_{0.4}O$ (PDF# 75-0273). The XRD pattern of Denary-MPOs/MCM-41 have complex diffraction peaks, belonging to multiphase metal oxides of cubic M_3O_4 (PDF# 74-1919), tetragonal MO_2 (PDF# 70-4177), cubic MO_2 (PDF# 89-8438), cubic MO (PDF# 71-1179).

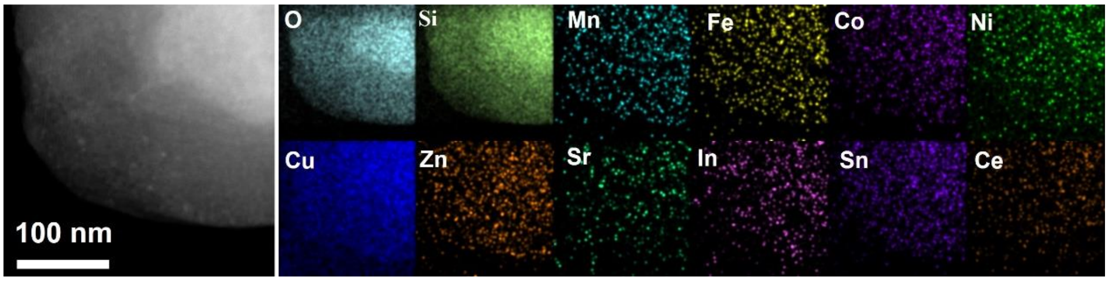

Supplementary Fig. 35: STEM images and elemental maps of Denary-HEOs@MCM-41.

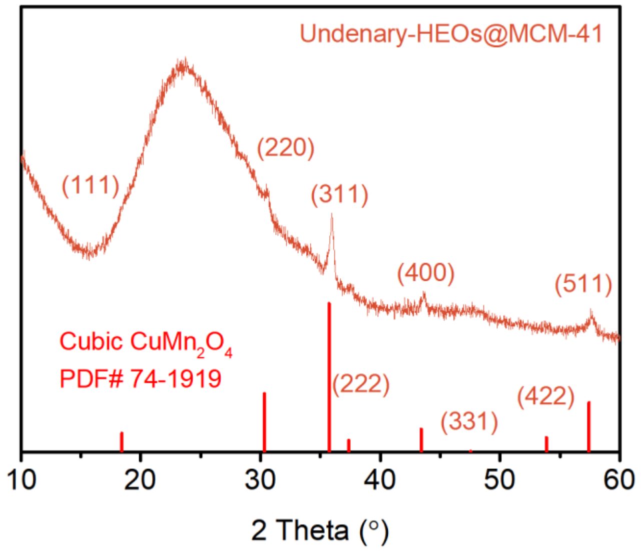

Supplementary Fig. 36: PXRD pattern of Undenary-HEOs@MCM-41 prepared by ICQ strategy.

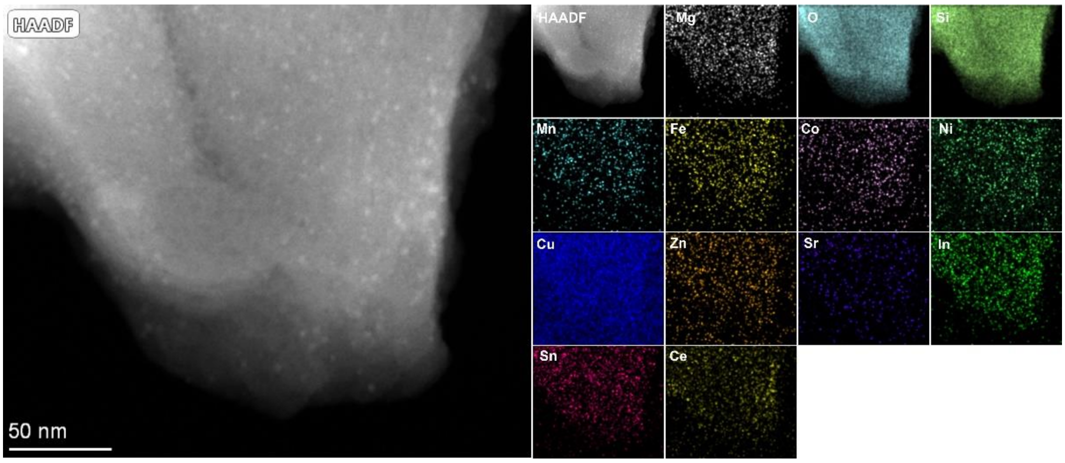

Supplementary Fig. 37: HAADF-STEM image and elemental maps of Undenary-HEOs@MCM-41.

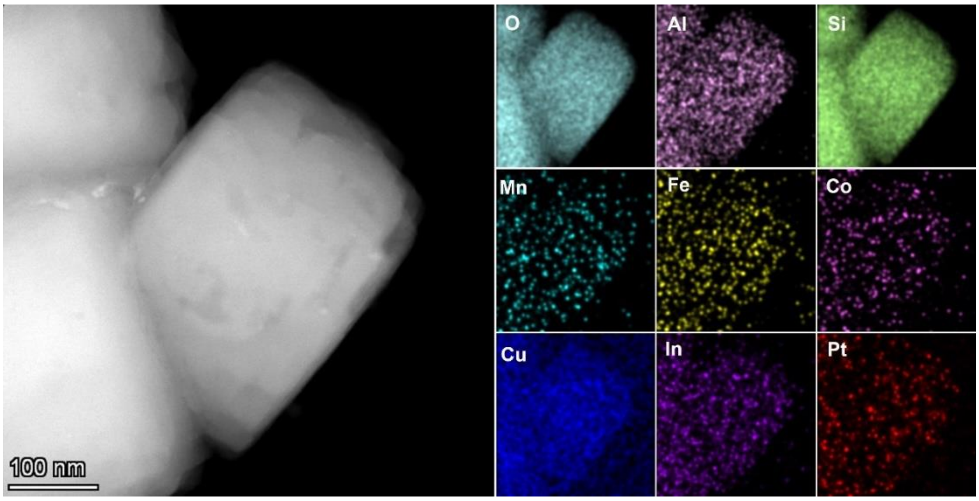

Supplementary Fig. 38: HAADF-STEM image and elemental maps of Pt-Quinary-HEOs@ZSM-5.

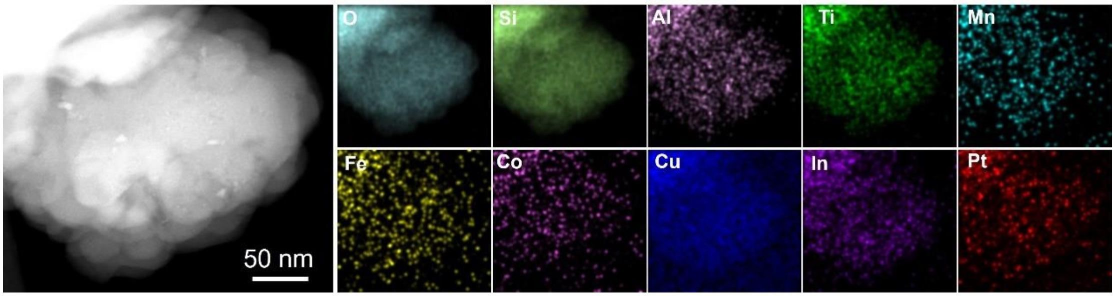

Supplementary Fig. 39: HAADF-STEM image and elemental maps of Pt-Quinary-HEOs@TS-1.

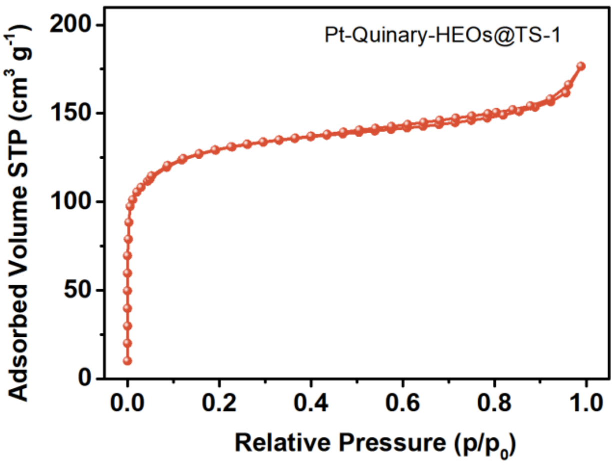

Supplementary Fig. 40: N_2 adsorption/desorption isotherm of Pt-Quinary-HEOs@TS-1.

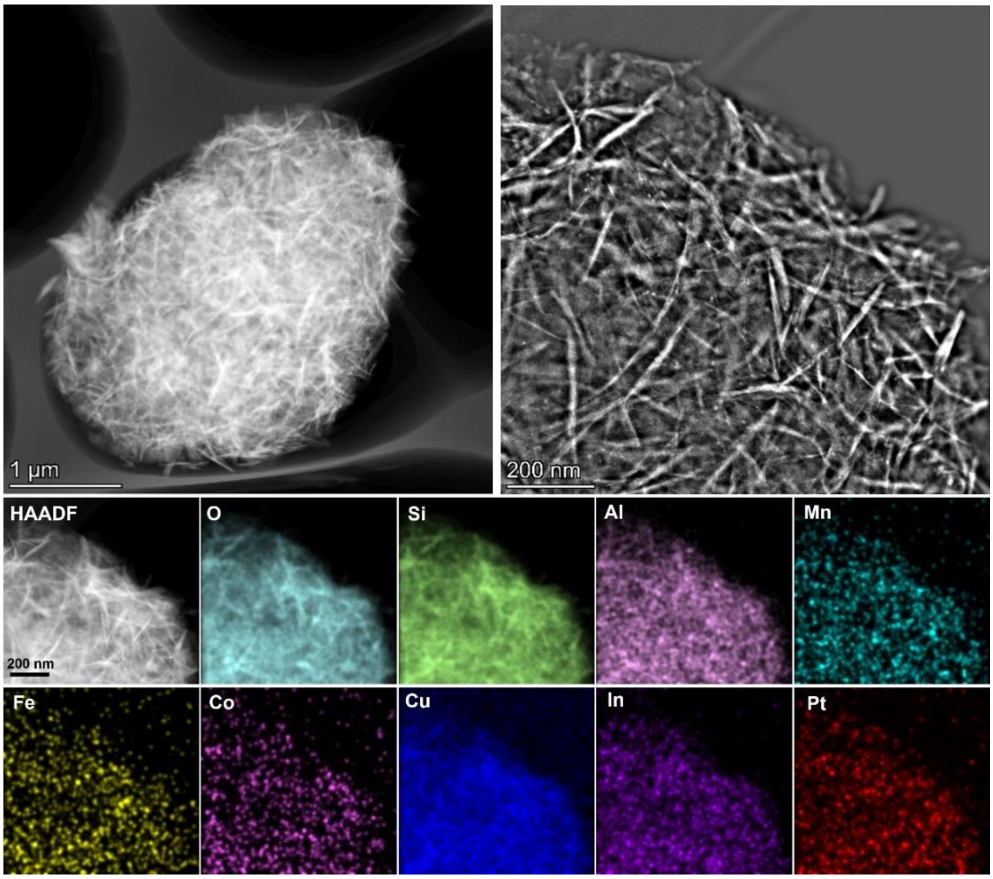

Supplementary Fig. 41: HAADF-STEM image and elemental maps of Pt-Quinary-HEOs@MCM-22.

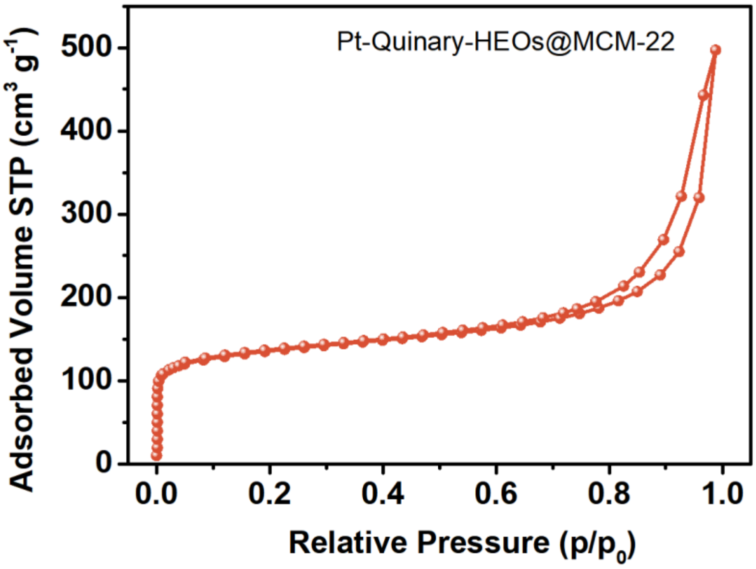

Supplementary Fig. 42: N_2 adsorption/desorption isotherm of Pt-Quinary-HEOs@MCM-22.

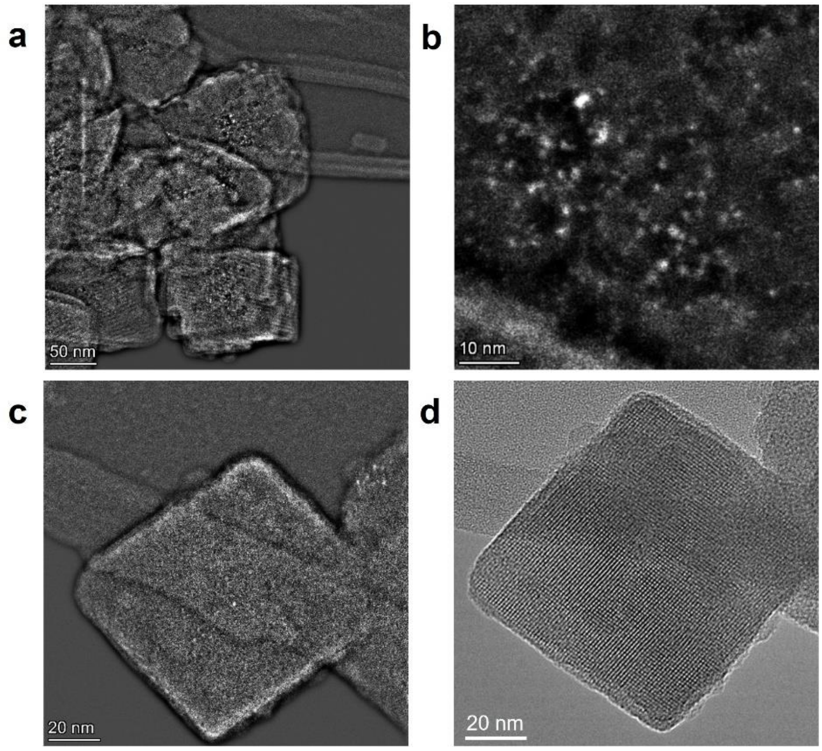

Supplementary Fig. 43: (a-c) HAADF-STEM and (d) HRTEM images of Pt-Quinary-HEOs@Beta.

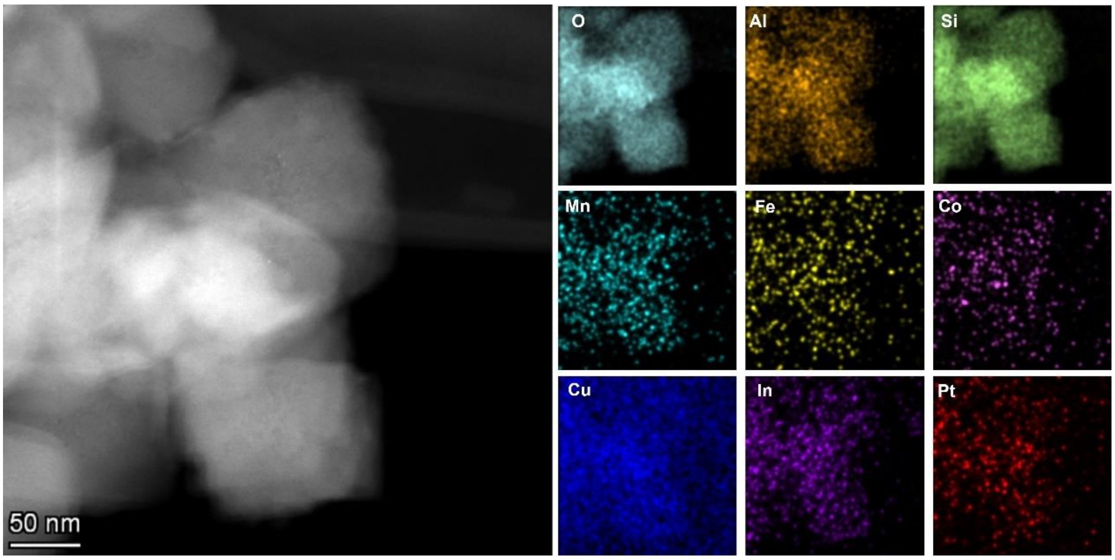

Supplementary Fig. 44: HAADF-STEM image and elemental maps of Pt-Quinary-HEOs@Beta.

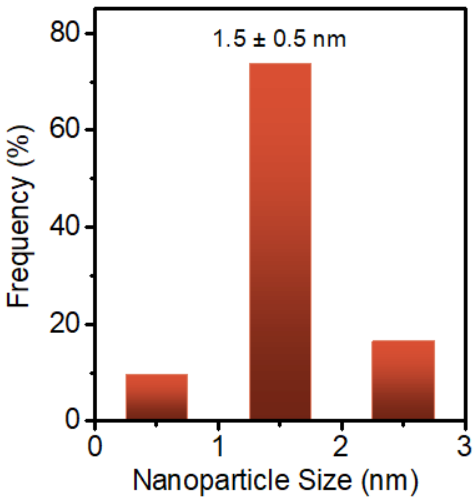

Supplementary Fig. 45: Size distributions of HEO nanoparticles of Pt-Quinary-HEOs@Beta.

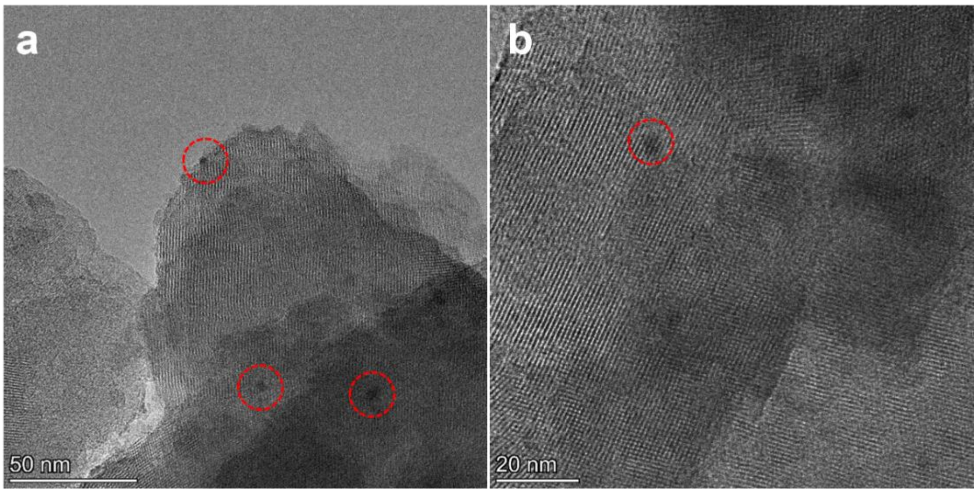

Supplementary Fig. 46: (a, b) HR-TEM images of Pd-Septenary-HEOs@Beta (The dark spots within the red circles are metal nanoparticles confined inside the zeolite particles).

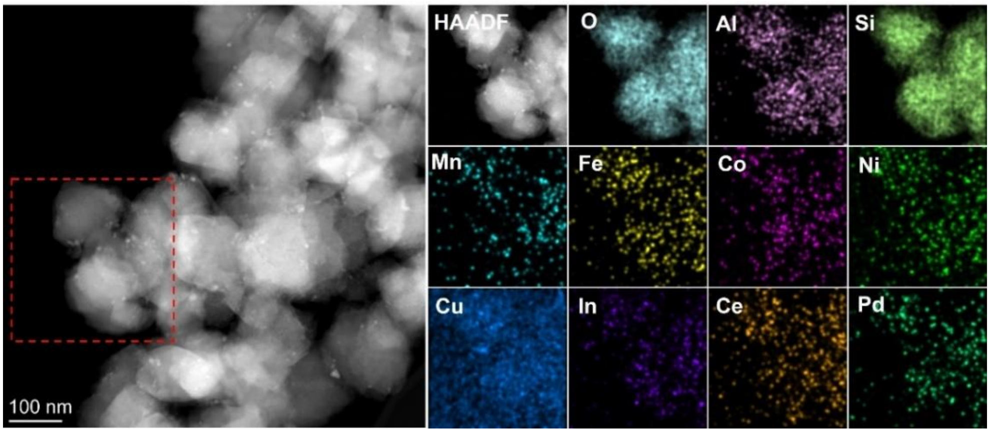

Supplementary Fig. 47: HAADF-STEM image and elemental maps of Pd-Septenary-HEOs@Beta.

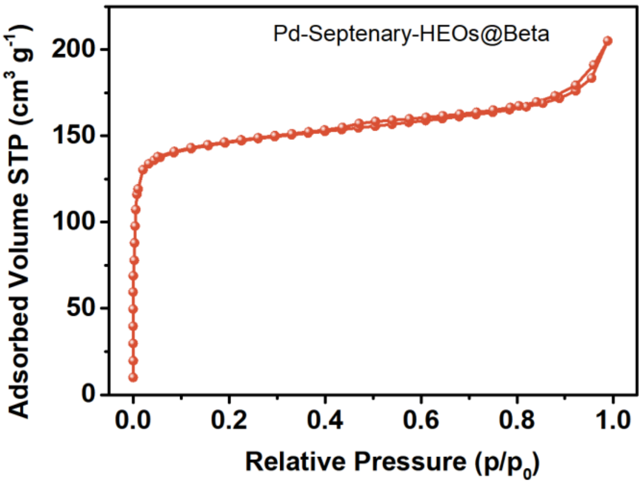

Supplementary Fig. 48: N_2 adsorption/desorption isotherm of Pt-Septenary-HEOs@Beta.

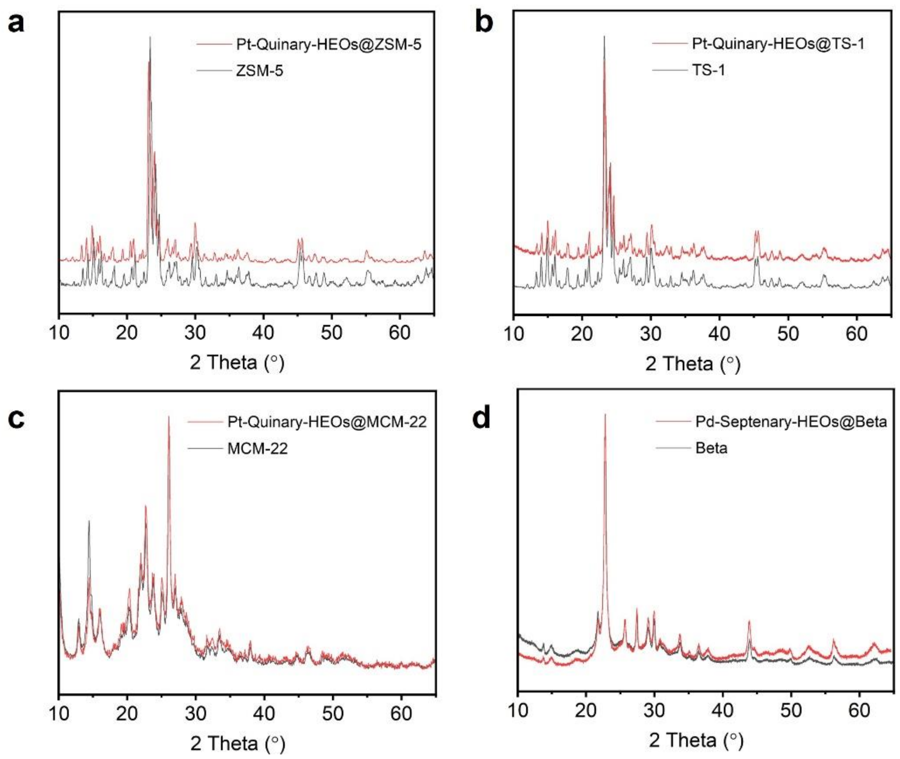

Supplementary Fig. 49: PXRD patterns of (a) Pt-Quinary-HEOs@ZSM-5, (b) Pt-Quinary-HEOs@TS-1, (c) Pt-Quinary-HEOs@MCM-22, (d) Pd-Septenary-HEOs@Beta prepared by ICQ strategy.

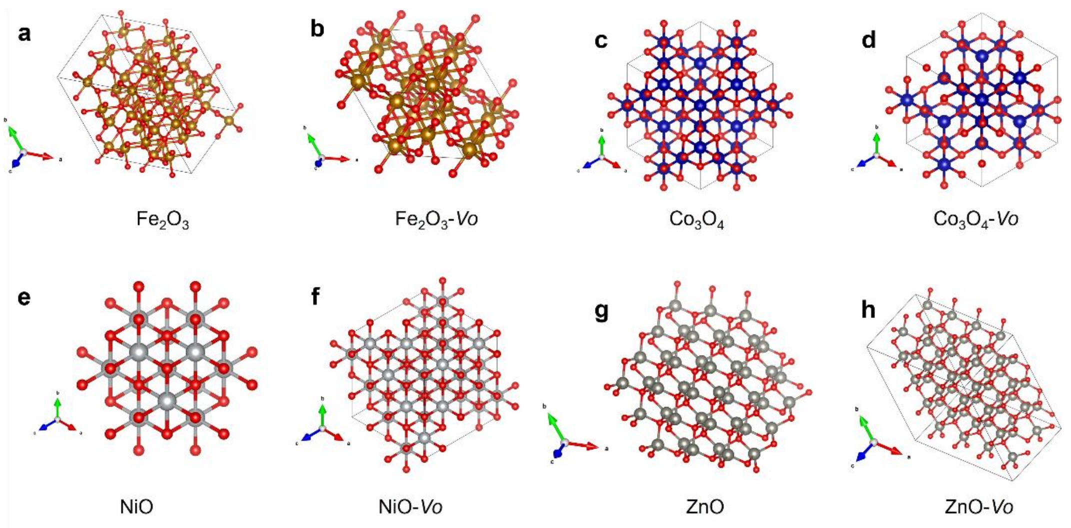

Supplementary Fig. 50: The models of (a) Fe_2O_3 and (b) Fe_2O_3-*Vo* ; (c) Co_3O_4 and (d) Co_3O_4-*Vo;* (e) NiO and (f) NiO-*Vo*; (g) ZnO and (h) ZnO-*Vo* used to calcinated the energy of oxygen vacancy formation.

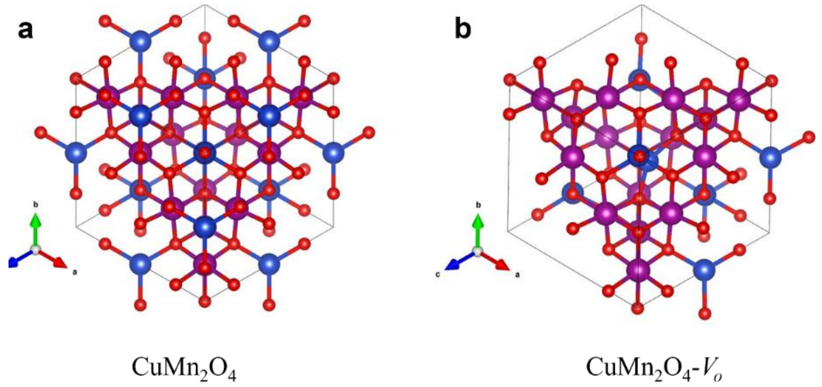

Supplementary Fig. 51: The models of (a) $CuMn_2O_4$ and (b) $CuMn_2O_4$-*Vo* used to calcinated the energy of oxygen vacancy formation.

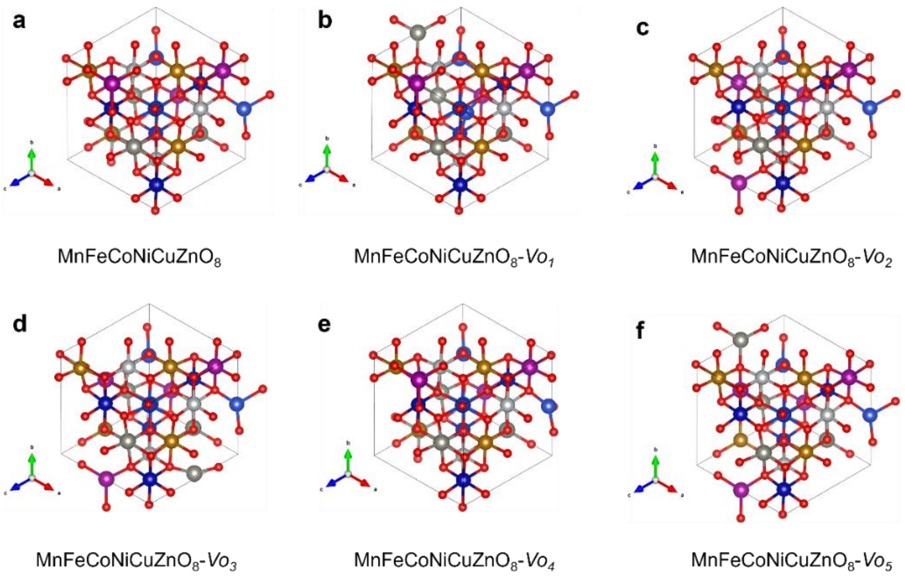

Supplementary Fig. 52: The models of (a) $MnFeCoNiCuZnO_8$, (b) $MnFeCoNiCuZnO_8$-Vo_1, (c) $MnFeCoNiCuZnO_8$-Vo_2, (d) $MnFeCoNiCuZnO_8$-Vo_3, (e) $MnFeCoNiCuZnO_8$-Vo_4, (f) $MnFeCoNiCuZnO_8$-Vo_5 used to calcinated the formation energy of oxygen vacancies with different chemical environments in high entropy oxides.

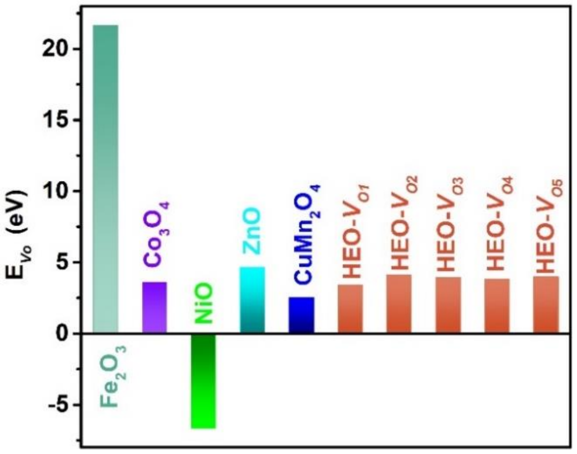

Supplementary Fig. 53: Oxygen vacancy formation energy (E_{Vo}) in single metal oxides, bimetallic oxides, and Senary-HEOs.

Notes: In high-entropy materials, multiple metal elements randomly occupy the same lattice regimes, leading to the emergence of unique physicochemical properties because of the complex elemental interactions. Reduction capability is one of the most significant features for metal oxides, which is determined by the chemical environment of oxygen species. First-principles calculations were carried out to study the oxygen vacancy formation energy (E_{Vo}) of single metal oxides, bimetallic oxides and HEOs.

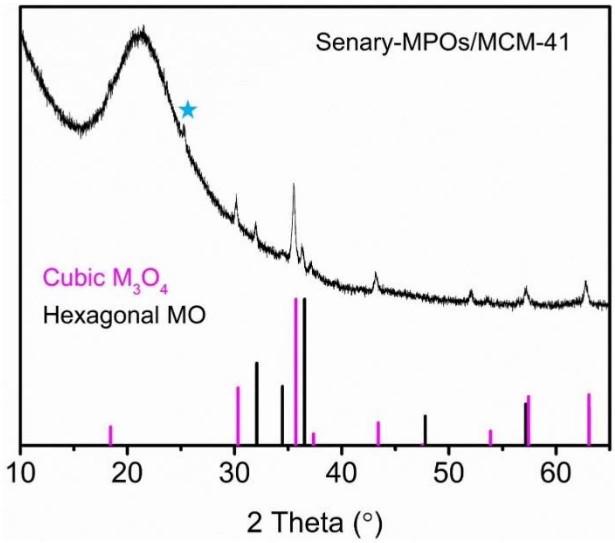

Supplementary Fig. 54: PXRD pattern of Senary-MPOs/MCM-41 prepared by initial wetness impregnation, calcination, and anneal process.

Notes: For the Senary-MPOs/MCM-41 sample prepared by incipient wetness impregnation, calcination, and anneal process, the peaks of XRD pattern are attributed to cubic M_3O_4 (PDF# 74-1919), hexagonal MO (PDF# 75-1526) and other impurity oxides (⋆).

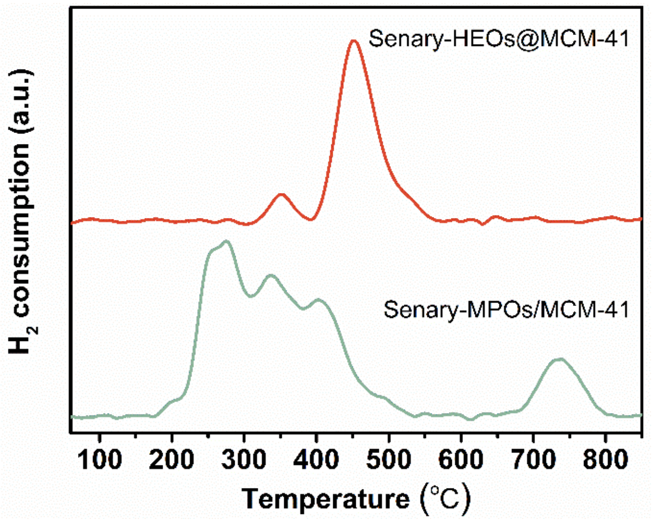

Supplementary Fig. 55: H_2-TPR profiles of Senary-HEOs@MCM-41 and Senary-MPOs/MCM-41.

Notes: The reducibility of as-prepared HEOs@MSs was performed by H_2-temperature-programmed reduction (H_2-TPR), compared with MPOs/MSs. The hydrogen consumption signal of Senary-HEOs@MCM-41 show a main peak at 450 °C with a weak satellite peak at 350 °C, which is associated with the release of lattice oxygen species and surface oxygen of Senary-HEO nanoparticles. In stark contrast, the H_2-TPR profile of Senary-MPOs/MCM-41 exhibits multiple reduction peaks in the range of 200-800 °C. These peaks are attributed to the distinct reduction behaviors of oxygen species present in the different metal oxides. These results demonstrate that each oxygen atom in the HEO nanoparticles loses its distinct identity as part of a mono-elemental oxide owing to the diverse configurations of neighboring metal atoms.

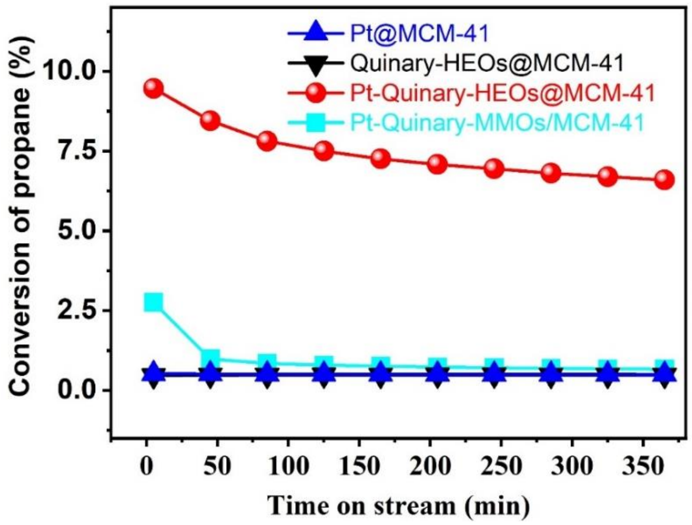

Supplementary Fig. 56: The conversion of propane over various catalysts. Reaction conditions: 0.1 g of catalyst mixed with 1.0 g of quartz sand, atmospheric pressure, C_3H_8/N_2 = 12.5/37.5 mL min^{-1}, WHSV = 13.5 h^{-1}, 550 °C.

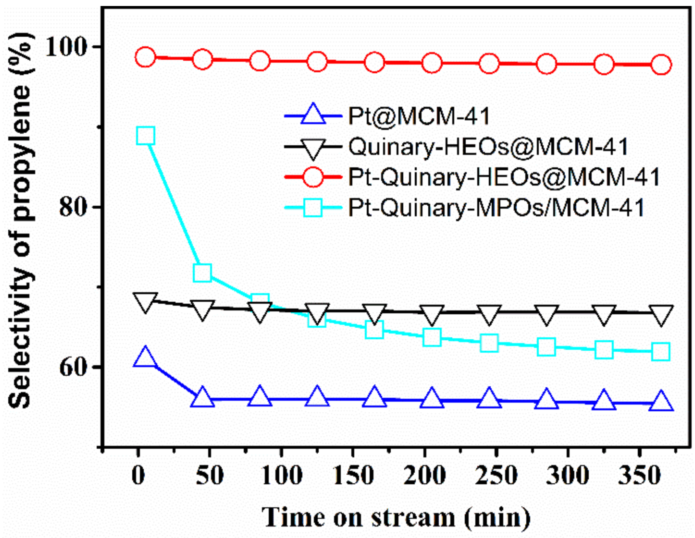

Supplementary Fig. 57: The propylene selectivity over various catalysts. Reaction conditions: 0.1 g of catalyst mixed with 1.0 g of quartz sand, atmospheric pressure, C_3H_8/N_2 = 12.5/37.5 mL min^{-1}, WHSV = 13.5 h^{-1}, 550 °C.

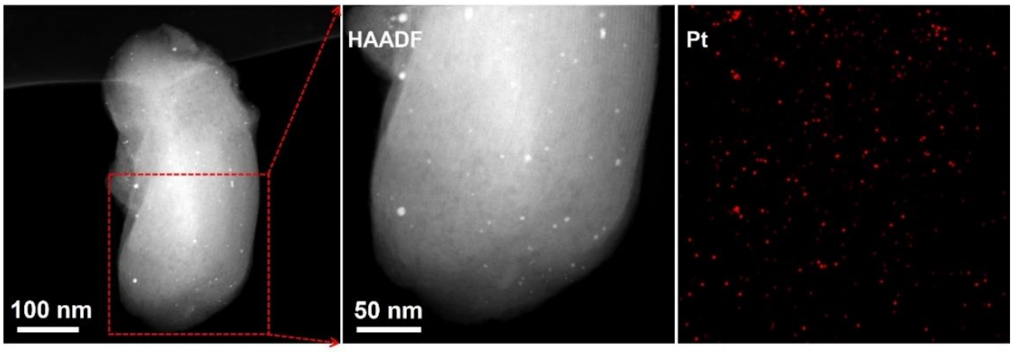

Supplementary Fig. 58: HAADF-STEM images of Pt@MCM-41, with the elemental map of Pt.

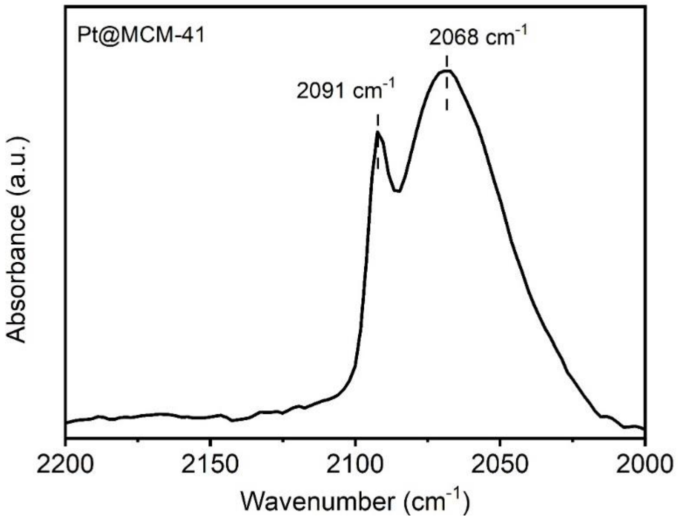

Supplementary Fig. 59: CO-DRIFTS profile of Pt@MCM-41.

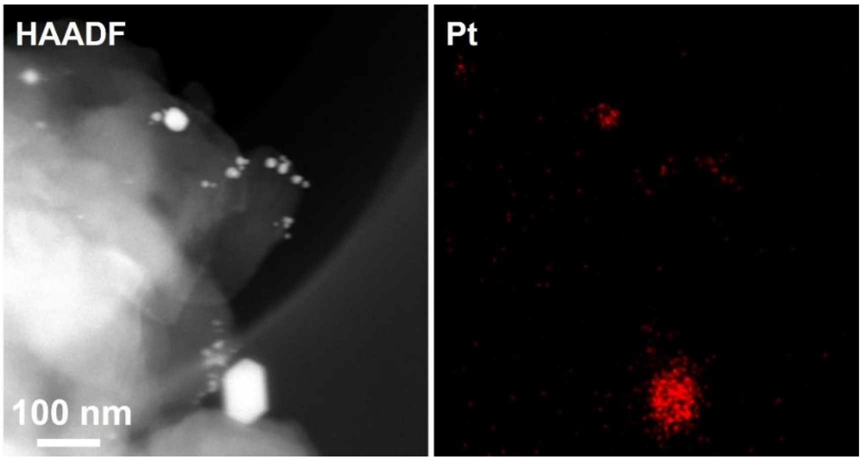

Supplementary Fig. 60: HAADF-STEM image of Pt/MCM-41, with the elemental map of Pt.

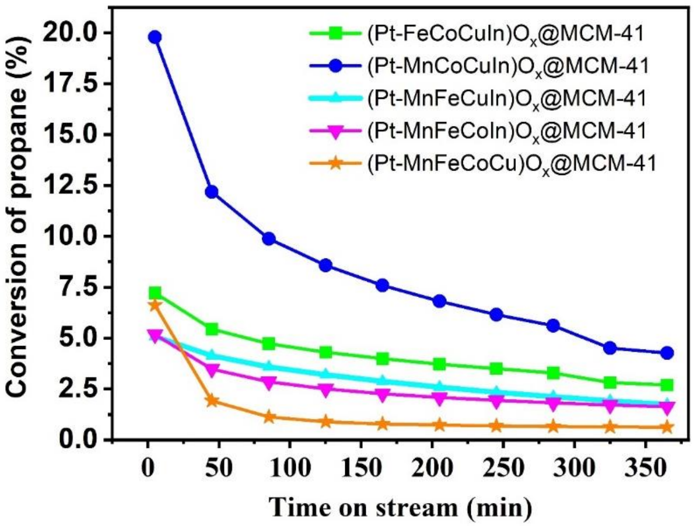

Supplementary Fig. 61: Effect of individual metal components of (Pt-MnFeCoCuIn)O_x@MCM-41 on PDH performance: Conversion of propane over various catalysts by removing one element at a time from the (Pt-MnFeCoCuIn)O_x@MCM-41. Reaction conditions: 0.1 g of catalyst mixed with 1.0 g of quartz sand, atmospheric pressure, C_3H_8/N_2 = 12.5/37.5 mL min^{-1}, WHSV = 13.5 h^{-1}, 550 °C.

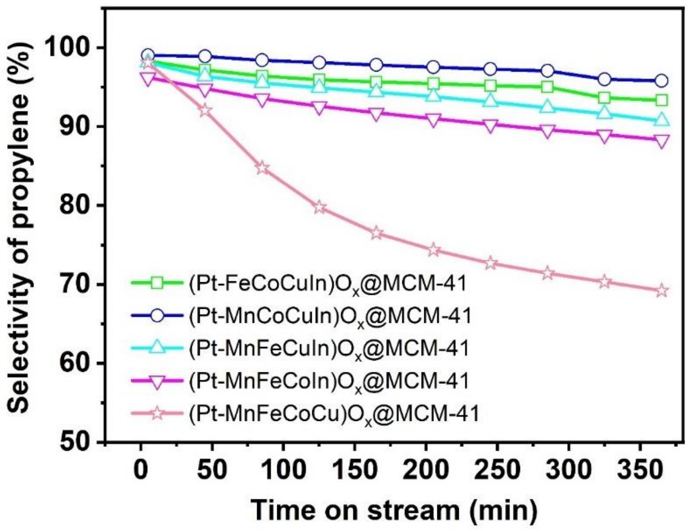

Supplementary Fig. 62: Effect of individual metal components of (Pt-MnFeCoCuIn)O_x@MCM-41 on PDH performance: Propylene selectivity over various catalysts by removing one element at a time from the (Pt-MnFeCoCuIn)O_x@MCM-41. Reaction conditions: 0.1 g of catalyst mixed with 1.0 g of quartz sand, atmospheric pressure, C_3H_8/N_2 = 12.5/37.5 mL min^{-1}, WHSV = 13.5 h^{-1}, 550 °C.

Notes: To elucidate the role of each metal in the high-entropy system, a series of catalysts were prepared by sequentially removing one element from the (Pt-MnFeCoCuIn)O_x@MCM-41 composition, and their performance in propane dehydrogenation was evaluated. The (Pt-MnCoCuIn)O_x@MCM-41 catalyst,

lacking Fe, exhibited an increased initial conversion rate of 19.8%, which declined sharply to 4.3% after 365 min, indicating that Fe suppresses initial activity but enhances long-term stability. In contrast, the performances of (Pt-FeCoCuIn)O_x@MCM-41, (Pt-MnCoCuIn)O_x@MCM-41, and (Pt-MnFeCoIn)O_x@MCM-41 showed that the absence of Mn, Co, or Cu led to lower initial conversions (5.0–7.3%), further declining to 1.5–2.5% over time, suggesting that these elements are essential for maintaining catalytic activity. The (Pt-MnFeCoCu)Ox@MCM-41 catalyst, with In removed, showed an initial conversion of 6.6%, which dropped drastically to 0.6% after 365 min. Additionally, the selectivity decreased markedly from approximately 90% to 68.5%, highlighting the pivotal role of In in preserving both activity and selectivity.

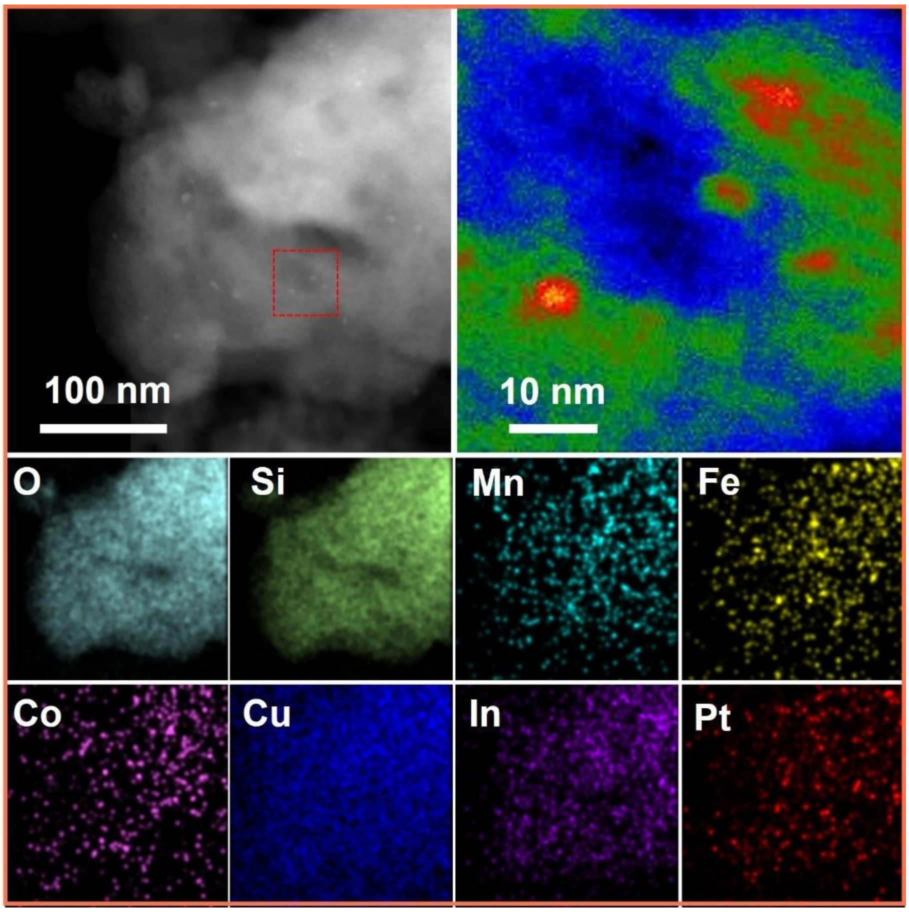

Supplementary Fig. 63: STEM images and EDS maps of Pt-Quinary-HEOs@MCM-41-spent.

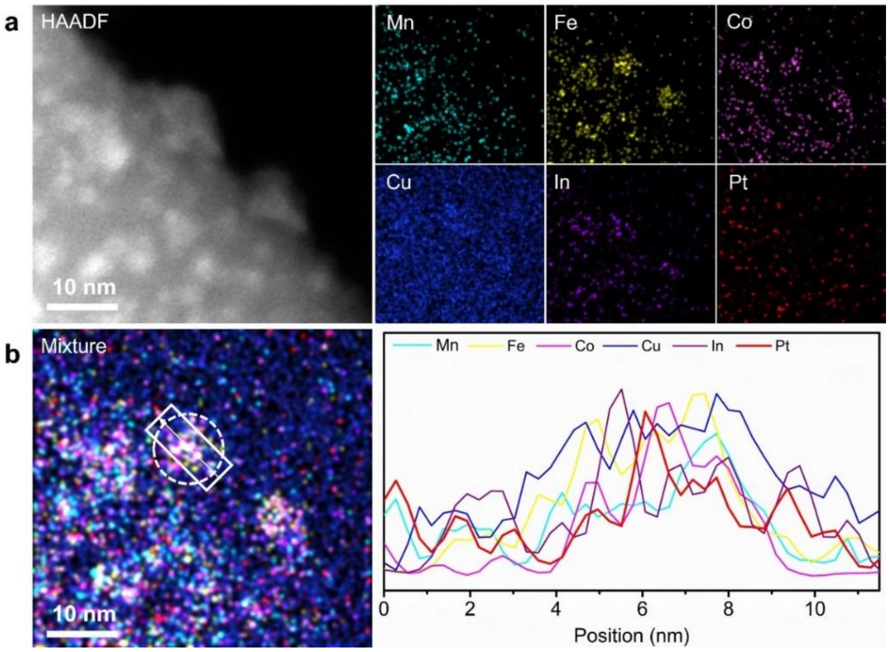

Supplementary Fig. 64: (a) Elemental mapping and (b) corresponding line scan profiles for nanoparticles of Pt-Quinary-HEOs@MCM-41-spent.

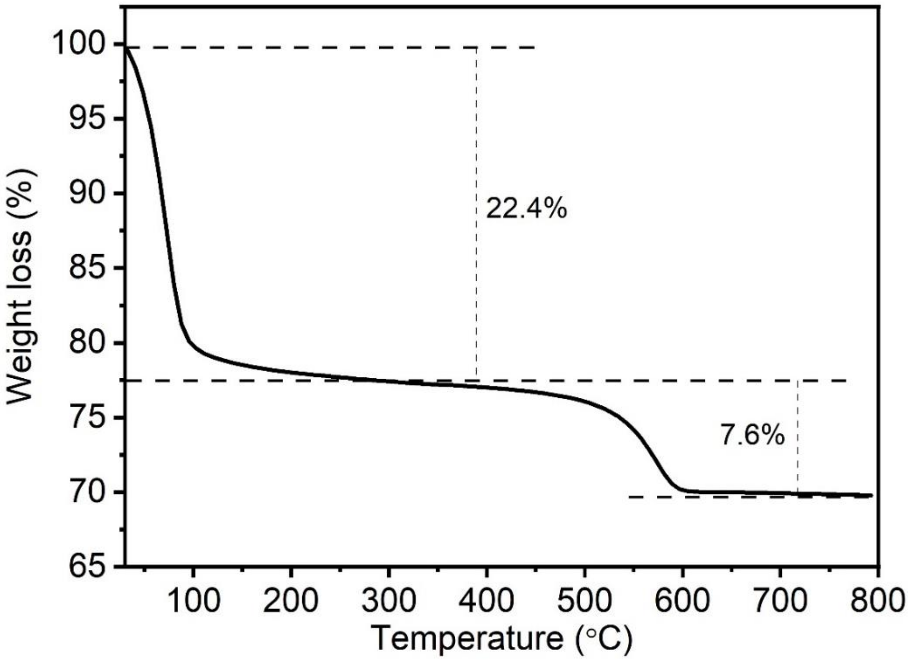

Supplementary Fig. 65: TGA of Pt-Quinary-HEOs@MCM-41-spent.

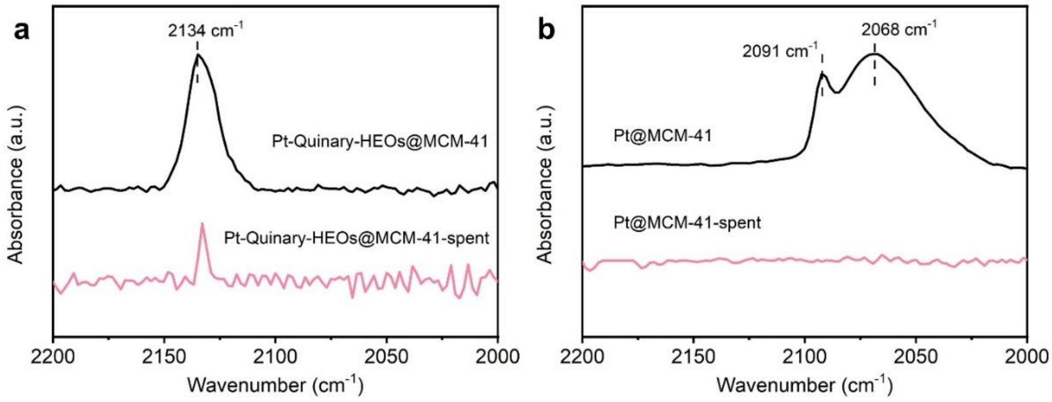

Supplementary Fig. 66: CO-DRIFTS profiles of (a) Pt-Quinary-HEOs@MCM-41, Pt-Quinary-HEOs@MCM-41-spent, and (b) Pt@MCM-41, Pt@MCM-41-spent.

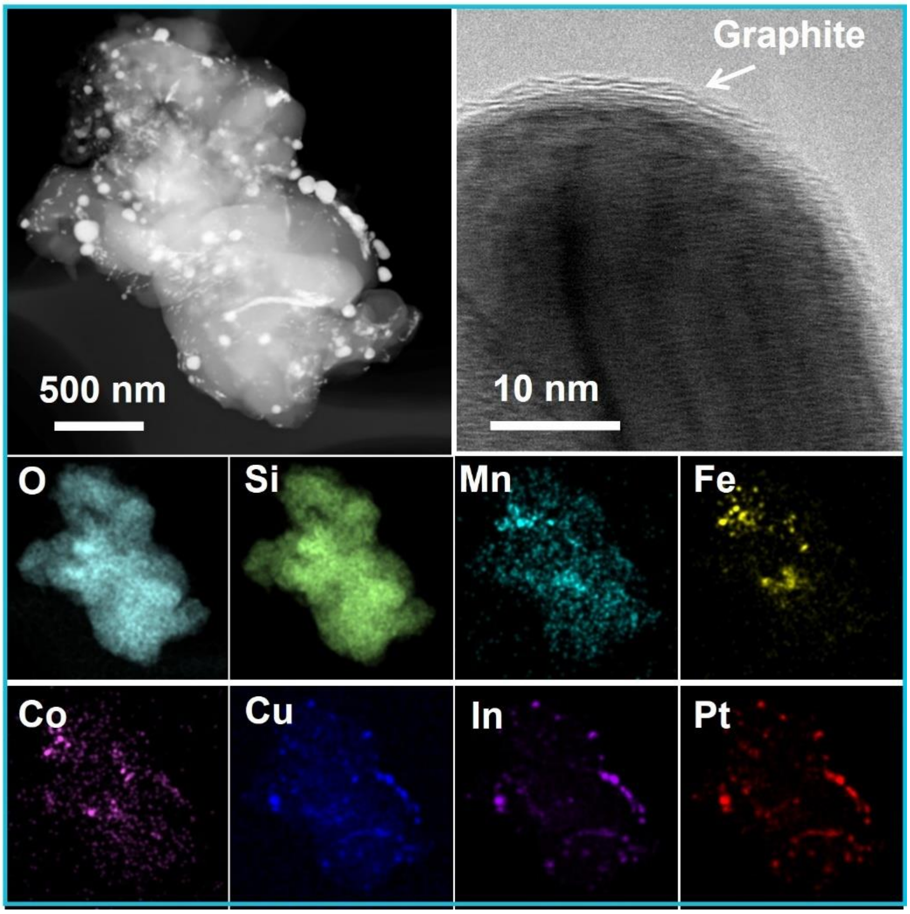

Supplementary Fig. 67: STEM images and EDS maps of Pt-Quinary-MPOs/MCM-41 after propane dehydrogenation testing.

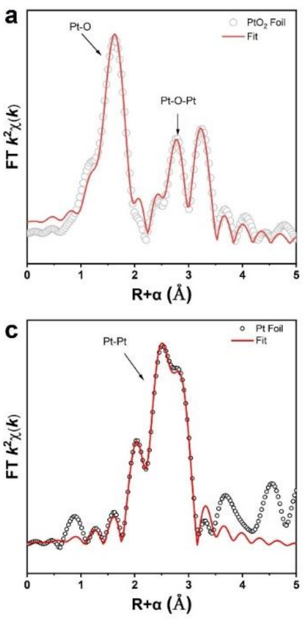

Supplementary Fig. 68: *R*-space of EXAFS and the fitting plots of (a) PtO_2 foil, (b) Pt-Quinary-HEOs@MCM-41 (fresh), (c) Pt-Quinary-HEOs@MCM-41 (spent) and (d) Pt foil.

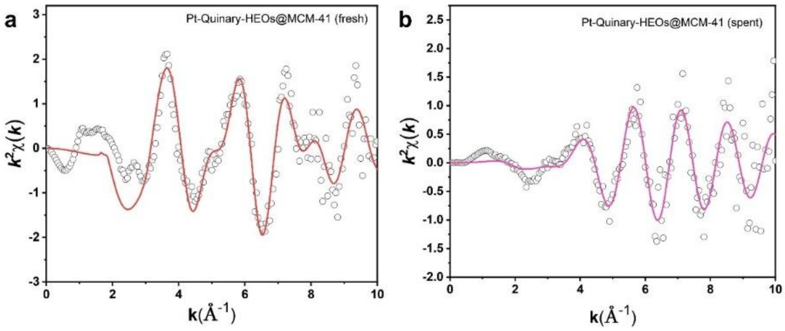

Supplementary Fig. 69: *K*-space of EXAFS and the fitting plots of (a) Pt-Quinary-HEOs@MCM-41(fresh) and (b) Pt-Quinary-HEOs@MCM-41 (spent).

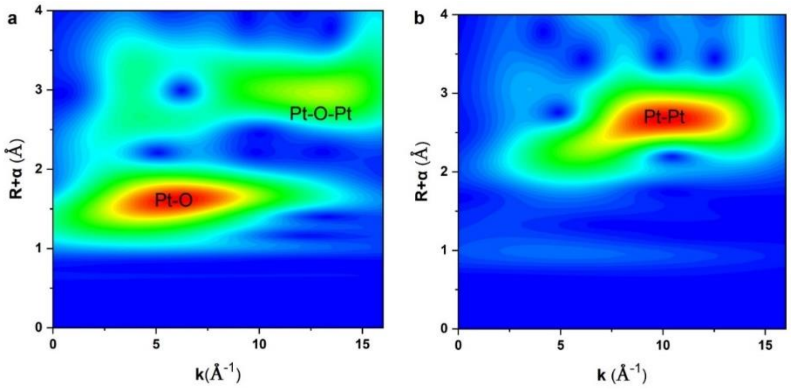

Supplementary Fig. 70: Wavelet-transformed EXAFS plots of (a) PtO_2 and (b) Pt foil.

Supplementary Table 1: Textural porosities of the MCM-41, Pt-Quinary-HEOs@MCM-41, Pt-Quinary-MPOs/MCM-41 samples.

Sample	S_{BET} (m^2/g)	V_{total} (cm^3/g)	Average pore diameter (nm)
MCM-41	1083.1	0.88	2.8
Pt-Quinary-HEOs@MCM-41	932.9	0.82	3.0
Pt-Quinary-MPOs/MCM-41	915.8	0.71	2.7

Supplementary Table 2: Textural properties of Pt-Senary-HEO@ZSM-5, Pt-Quinary-MPOs/NaY, Pt-Quinary-HEOs@HY, Pt-Quinary-HEOs@TS-1, Pt-Quinary-HEOs@MCM-22, Pd-Septenary-HEOs@Beta.

Sample	$^{a}S_{BET}$ (m^2/g)	$^{b}S_{Micro}$ (m^2/g)	$^{b}S_{ext}$ (m^2/g)	$^{b}V_{Micro}$ (cm^3/g)	$^{c}S_{mes}$ (cm^3/g)
Pt-Senary-HEO@ZSM-5	299	117	182	0.06	0.14
Pt-Quinary-MPOs/NaY	701	625	76	0.32	0.07
Pt-Quinary-HEOs@HY	618	528	90	0.28	0.13
Pt-Quinary-HEOs@TS-1	398	248	150	0.13	0.11
Pt-Quinary-HEOs@MCM-22	424	241	183	0.13	0.62
Pd-Septenary-HEOs@Beta	451	351	100	0.18	0.12

[a] S_{BET} (total surface area) calculated by applying the BET equation using the linear part (0.05 < P/P0< 0.30) of the adsorption isotherm. [b] S_{micro} (micropore area), S_{ext} (external surface) and Vmicro (micropore volume) calculated using the t-plot method. [c] V_{mes} (mesopore volume) calculated using the BJH method (from desorption).

Supplementary Table 3: Particle size of high-entropy-materials.

**Synthesis method**	**High-entropy material**	**Particle size (nm)**	**References**
ICQ Strategy	HEO@MCM-22	1.4 ± 0.2	This work
	HEO@Beta	1.5 ± 0.5	This work
	HEO@ZSM-5	1.6 ± 0.4	This work
	HEO@TS-1	2.8 ± 0.6	This work
	HEO@M41	3 ± 0.5	This work
	HEO@beta	4.0 ± 0.8	This work
	HEO@Beta	4.7 ± 1.1	This work

Wet chemical method	HEA	1.0	(12)
	HEO	1.0	(13)
	HEA	1.3 ± 0.4	(14)
	HEA	1.5	(15)
	HEA	1.7	(16)
	HEA	3.1 ± 0.6	(17)
	HEA	3.4 ± 0.6	(18)
	HEA	4.0 ± 0.9	(14)
	HEA	4.1 ± 1.2	(19)
	HEA	11 ± 4.6	(20)
	HEO	30.0	(20)
	HEO	32.8 ± 13.6	(21)
	HEA	35.0 ± 20.0	(22)
	HEA	56.2 ± 20.7	(20)
Calcination method	HEA	2.0	(23)
	HEO	4.0 ± 1.0	(24)
	HEA/Zeolite	5.0	(23)
	HEA	10.0	(25)
	HEA	133.0 ± 6.7	(26)
	HEO	3000	(27)
	HEO	10000	(28)
Thermal shock method	HEA/C	5.0	(29)
	HEA/C	5.3 ± 1.3	(30)
	HEA	10.0	(25)
	HEA/C	12.8 ± 5.1	(31)
	HEA/C	13.0 ± 2.0	(32)
	HEA	20.0	(33)
	HEO/C	20.0	(29)
	HEA	30.0	(34)
	HEA/C	38.5 ± 20.4	(31)
	HEA/C	160.0 ± 5.0	(35)
	HEO/C	184 ± 15.0	(35)

Supplementary Table 4: The formation energy of oxygen vacancies with different chemical environments in metal oxides and high entropy oxide of $(MnFeCoNiCuZn)O_8$.

**Metal oxide**	**The energy of oxygen vacancy formation (eV)**	**Chemical environment around oxygen vacancies**
Fe_2O_3	21.645	Fe
Co_3O_4	3.621	Co
NiO	-6.678	Ni
ZnO	4.632	Zn
$CuMn_2O_4$	2.533	MnMnMnCu
$MnFeCoNiCuZnO_8$-Vo_1	3.419	MnCoNiCu
$MnFeCoNiCuZnO_8$-Vo_2	4.113	MnFeCoNi
$MnFeCoNiCuZnO_8$-Vo_3	3.933	MnFeNiZn
$MnFeCoNiCuZnO_8$-Vo_4	3.825	MnFeCoCu
$MnFeCoNiCuZnO_8$-Vo_5	3.984	MnFeCoZn

Supplementary Table 5: Comparison of PDH activity among various heterogeneous noble metal-based catalysts.

Catalyst	Pt Loading (wt %)	T [ºC]	WHSV [h^{-1}]	Feed composition	Con. [%]	Sel. [%]	C_3H_6 formation rate [mol g_{Pt}^{-1} h^{-1}]	Ref.
**Pt-Quinary-HEOs@MCM-41**	0.065	550	13.5	$C_3H_8/N_2 = 25/75$	9.5	98.7	44.17	This work
**Pt-Quinary-MPOs/MCM-41**	0.063	550	13.5	$C_3H_8/N_2 = 25/75$	2.2	88.9	9.50	This work
**Pt@MCM-41**	0.069	550	13.5	$C_3H_8/N_2 = 25/75$	0.5	61.0	1.41	This work
**PtCuSnAuPd/SiO$_2$**	0.5	550		$C_3H_8/Ar = 25/75$	41.4	96.3	10.7	(36)
**PtLa/mz-deGa**	1	580	11	C_3H_8	42	95	7.34	(37)
**PtSnAl0.2/SBA-15**	0.44	590	2.5	$C_3H_8/Ar = 1/5$	55.9	98.5	7.11	(38)
**PtSn/2Mg-SBA-15**	0.86	580	8.25	$C_3H_8/Ar = 7/3$	43	95	9.17	(39)
**Leached PtFe@Pt/SBA-15**	0.75	600	6.9	$C_3H_8/H_2/N_2 = 26/26/48$	35	85	3.14	(40)
**PtSn/PZ**	0.71	550	3.6	$C_3H_8/N_2 = 25/75$	47.2	95.8	5.22	(41)
**PtSn/PZ**	0.71	550	27	$C_3H_8/N_2 = 25/75$			33.4	(41)
**Pt-Sn/CeO$_2$**	1.0	550	2.159	$C_3H_8/He = 2/10$	39.5	84.5	1.64	(42)
**0.04Pt-0.36Zn-DeAlBEA**	0.04	600	1.2	$C_3H_8/He = 25/75$	41	99.5	2.48	(43)
**Pt/In-ZSM-5**	1	580	118	$C_3H_8/Ar = 20/80$	20	99.8	49.1	(44)
**0.5PtHEA/SiO$_2$**	0.5	550	3.54	$C_3H_8/H_2/N_2 =$	36.4	96.7	5.61	(45)

				3/3/24				
**0.5PtHEA/SiO$_2$**	0.5	600	11.4	$C_3H_8/H_2/N_2$ = 3/3/24	35.5	97.9	21.8	(45)
**GaPt/SiO$_2$**	4.37	550	98	C_3H_8/Ar = 20/80	31.9	99	16.4	(46)
**PtGe/Ga-SiO$_2$**	1	600	2	C_3H_8/H_2/He= 2.5:1.3:3.7	33	98	0.68	(47)
**PtMn/SiO$_2$**	2.97	550	66.7	C_3H_8/Ar = 1/4	18.1	97.3	8.86	(48)
**K-PtSn@MFI**	0.42	600	1.7	C_3H_8/N_2 = 5/16	74	88	5.75	(49)
**K-PtSn@MFI-600 H$_2$-22h**	0.4	550	118.1	C_3H_8	20	>97	125.9	
**PtZn$_4$@S-1-H**	0.72	550	3.6	C_3H_8/N_2 = 1/3	47.7	93.2	4.93	(50)
**PtZn$_4$@S-1-H**	0.72	550	108	C_3H_8/N_2 = 1/3	21.2	98.3	65.5	(50)
**PtZn$_4$@S-1-H**	0.72	600	3.6	C_3H_8/N_2 = 1/3	66.7	90.8	6.88	(50)
**0.3Pt0.5Zn@S-1**	0.3	550	6.5	C_3H_8/N_2 = 11/19	45	98.0	21.5	(51)
**0.3Pt0.5Zn@S-1**	0.3	550	6.5	C_3H_8/N_2 = 11/19	40	99.0	28.2	(51)
**Pt/0.5Mn-DMSN**	-	520	9.4	C_3H_8/N_2 = 1/2	56.1	95.6	8.1	(52)
**Pt/0.5Mn-DMSN**	-	590	2.4	C_3H_8/N_2 = 1/2	56.1	95.6	5.8	(52)
**In/Rh0.01%@S-1**	0.01	550	150	C_3H_8	< 10	~ 90	1860	(53)

Supplementary Table 6: EXAFS fitting parameters at the Pt L_3-edge for various samples (S_0^2=0.79 from Pt-foil).

	shell	CN[a]	R[b] (Å)	σ^{2c} (Å^2)	ΔE_0^d(eV)	R factor
Pt-foil	Pt-Pt	12	2.76±0.01	0.0044	7.0±0.4	0.0035
PtO_2-foil	Pt-O	6.0±0.3	2.02±0.01	0.0030	9.4±0.8	0.0158
	Pt-O-Pt	6.1±0.5	3.10±0.01	0.0028		
	Pt-O	12.4±2.8	3.67±0.03	0.0107		
Pt-Quinary-HEOs@MCM-41	Pt-O	5.4±0.4	2.02±0.01	0.0030	10.2±3.0	0.0186
	Pt-O-M	10.1±1.2	3.06±0.02	0.0124		
Pt-Quinary-HEOs@MCM-41 (spent)	Pt-O	0.5±0.3	1.94±0.01	0.0060	-1.5±1.0	0.0097
	Pt-M	5.1±0.2	2.56±0.01	0.0062		

[a]*CN*: coordination numbers; [b]*R*: bond distance; [c]σ^2: Debye-Waller factors; [d] ΔE_0: the inner potential correction. R factor: goodness of fit. A reasonable range of EXAFS fitting parameters: $0.70 < S_0^2 < 1.00$; CN > 0; $\sigma^2 > 0$ Å^2; $|\Delta E_0| < 15$ eV; R factor < 0.02.

Supporting References

1. Wang, Y., C. Shu, H. B. Huang, C. J. Teo, Multiphase lattice Boltzmann flux solver for incompressible multiphase flows with large density ratio. *J. Comput. Phys* **280**, 404-423 (2015).
2. Li, X., P. Yu, X.-D. Niu, D.-C. Li, H. Yamaguchi, A magnetic field coupling lattice Boltzmann model and its application on the merging process of multiple-ferrofluid-droplet system. *Appl. Math. Comput.* **393**, 125769 (2021).
3. Zheng, H. W., C. Shu, Y. T. Chew, A lattice Boltzmann model for multiphase flows with large density ratio. *J. Comput. Phys.* **218**, 353-371 (2006).
4. Niu, X.-D. *et al.*, A mass-conserving multiphase lattice Boltzmann model for simulation of multiphase flows. *Phys. Fluids* **30**, 013302 (2018).
5. Li, X. *et al.*, A fractional-step lattice Boltzmann method for multiphase flows with complex interfacial behavior and large density contrast. *Int. J. Multiphas. Flow* **149**, 103982 (2022).
6. Li, X. *et al.*, A magnetic field coupling fractional step lattice Boltzmann model for the complex interfacial behavior in magnetic multiphase flows. *Appl. Math. Model.* **117**, 219-250 (2023).
7. Kresse, G., D. Joubert, From ultrasoft pseudopotentials to the projector augmented-wave method. *Phys. Rev. B* **59**, 1758 (1999).
8. Blöchl, P. E., O. Jepsen, O. K. Andersen, Improved tetrahedron method for Brillouin-zone integrations. *Phys. Rev. B* **49**, 16223 (1994).
9. Blöchl, P. E., Projector augmented-wave method. *Phys. Rev. B* **50**, 17953 (1994).
10. Kresse, G., J. Furthmüller, Efficient iterative schemes for ab initio total-energy calculations using a plane-wave basis set. *Phys. Rev. B* **54**, 11169 (1996).
11. Monkhorst, H. J., J. D. Pack, Special points for Brillouin-zone integrations. *Phys. Rev. B* **13**, 5188 (1976).
12. Sun, Y. *et al.*, A general approach to high-entropy metallic nanowire electrocatalysts. *Matter* **6**, 193-205 (2023).
13. Liu, J. *et al.*, Polyoxometalate Cluster-Incorporated High Entropy Oxide Sub-1 nm Nanowires. *J. Am. Chem. Soc.* **144**, 23191-23197 (2022).
14. Minamihara, H. *et al.*, Continuous-Flow Reactor Synthesis for Homogeneous 1 nm-Sized Extremely Small High-Entropy Alloy Nanoparticles. *J. Am. Chem. Soc.* **144**, 11525-11529 (2022).
15. Tao, L. *et al.*, A General Synthetic Method for High-Entropy Alloy Subnanometer Ribbons. *J. Am. Chem. Soc.* **144**, 10582-10590 (2022).
16. Feng, G. *et al.*, Sub-2 nm Ultrasmall High-Entropy Alloy Nanoparticles for Extremely Superior Electrocatalytic Hydrogen Evolution. *J. Am. Chem. Soc.* **143**, 17117-17127 (2021).
17. Wu, D. *et al.*, Platinum-Group-Metal High-Entropy-Alloy Nanoparticles. *J. Am. Chem. Soc.* **142**, 13833-13838 (2020).
18. Li, H. *et al.*, Fast site-to-site electron transfer of high-entropy alloy nanocatalyst driving redox electrocatalysis. *Nat. Commun.* **11**, 5437 (2020).
19. Wu, D. *et al.*, Noble-Metal High-Entropy-Alloy Nanoparticles: Atomic-Level Insight into the Electronic Structure. *J. Am. Chem. Soc.* **144**, 3365-3369 (2022).

20. Wang, B. *et al.*, General synthesis of high-entropy alloy and ceramic nanoparticles in nanoseconds. *Nat. Synth.* **1**, 138-146 (2022).
21. Hanabata, S. *et al.*, Denary High-Entropy Oxide Nanoparticles Synthesized by a Continuous Supercritical Hydrothermal Flow Process. *J. Am. Chem. Soc.* **146**, 181-186 (2024).
22. He, R. *et al.*, A 3d-4d-5d High Entropy Alloy as a Bifunctional Oxygen Catalyst for Robust Aqueous Zinc-Air Batteries. *Adv. Mater.* **35**, 2303719 (2023).
23. Gao, S. *et al.*, Synthesis of high-entropy alloy nanoparticles on supports by the fast moving bed pyrolysis. *Nat. Commun.* **11**, 2016 (2020).
24. Miao, K. *et al.*, Hollow-Structured and Polyhedron-Shaped High Entropy Oxide toward Highly Active and Robust Oxygen Evolution Reaction in a Full pH Range. *Adv. Mater.*, 2308490 (2023).
25. Fan, L. *et al.*, High Entropy Alloy Electrocatalytic Electrode toward Alkaline Glycerol Valorization Coupling with Acidic Hydrogen Production. *J. Am. Chem. Soc.* **144**, 7224-7235 (2022).
26. Liang, J. *et al.*, High-Entropy Alloy Array via Liquid Metal Nanoreactor. *Adv. Mater.* **36**, 2403865 (2024).
27. Wang, Y. *et al.*, An Unexpected Decrease in Vibrational Entropy of Multicomponent Rutile Oxides. *J. Am. Chem. Soc.* **146**, 14493-14504 (2024).
28. Rost, C. M. *et al.*, Entropy-stabilized oxides. *Nat. Commun.* **6**, 8485 (2015).
29. Zheng, X. *et al.*, Hydrogen-substituted graphdiyne-assisted ultrafast sparking synthesis of metastable nanomaterials. *Nat. Nanotech.* **18**, 153-159 (2023).
30. Yao, Y. *et al.*, Carbothermal shock synthesis of high-entropy-alloy nanoparticles. *Science* **359**, 1489-1494 (2018).
31. Qiao, H. *et al.*, Scalable Synthesis of High Entropy Alloy Nanoparticles by Microwave Heating. *ACS Nano* **15**, 14928-14937 (2021).
32. Wang, X. *et al.*, Continuous Synthesis of Hollow High-Entropy Nanoparticles for Energy and Catalysis Applications. *Adv. Mater.* **32**, 2002853 (2020).
33. Huang, Z. *et al.*, Tailoring Local Chemical Ordering via Elemental Tuning in High-Entropy Alloys. *J. Am. Chem. Soc.* **146**, 2167-2173 (2024).
34. Chen, Z. W. *et al.*, Unusual Sabatier principle on high entropy alloy catalysts for hydrogen evolution reactions. *Nat. Commun.* **15**, 359 (2024).
35. Wang, X. *et al.*, Continuous 2000 K droplet-to-particle synthesis. *Mater. Today* **35**, 106-114 (2020).
36. Luo, J. *et al.*, Progressive Fabrication of a Pt-Based High-Entropy-Alloy Catalyst toward Highly Efficient Propane Dehydrogenation. *Angew. Chem. Int. Ed.* **64**, e202419093 (2025).
37. Ryoo, R. *et al.*, Rare-earth-platinum alloy nanoparticles in mesoporous zeolite for catalysis. *Nature* **585**, 221-224 (2020).
38. Fan, X. Q. *et al.*, Dehydrogenation of propane over PtSnAl/SBA-15 catalysts: Al addition effect and coke formation analysis. *Catal. Sci. Technol.* **5**, 339-350 (2015).
39. Li, B., Z. X. Xu, F. L. Jing, S. Z. Luo, W. Chu, Facile one-pot synthesized ordered mesoporous Mg-SBA-15 supported PtSn catalysts for propane dehydrogenation. *Appl. Catal. A-Gen.* **533**, 17-27 (2017).

40. Cai, W. T. *et al.*, Subsurface catalysis-mediated selectivity of dehydrogenation reaction. *Sci. Adv.* **4**, eaar5418 (2018).
41. Li, J. *et al.*, Silanol-Stabilized Atomically Dispersed $Pt^{\delta+}$-O_x-Sn Active Sites in Protozeolite for Propane Dehydrogenation. *J. Am. Chem. Soc.* **146**, 24358-24367 (2024).
42. Xiong, H. F. *et al.*, Thermally Stable and Regenerable Platinum-Tin Clusters for Propane Dehydrogenation Prepared by Atom Trapping on Ceria. *Angew. Chem. Int. Ed.* **56**, 8986-8991 (2017).
43. Qi, L. *et al.*, Propane Dehydrogenation Catalyzed by Isolated Pt Atoms in ≡SiOZn-OH Nests in Dealuminated Zeolite Beta. *J. Am. Chem. Soc.* **143**, 21364-21378 (2021).
44. Yuan, Y., E. Huang, S. Hwang, P. Liu, J. G. Chen, Confining platinum clusters in indium-modified ZSM-5 zeolite to promote propane dehydrogenation. *Nat. Commun.* **15**, 6529 (2024).
45. Zhou, S.-Z. *et al.*, An Active and Regenerable Nanometric High-Entropy Catalyst for Efficient Propane Dehydrogenation. *Angew. Chem. Int. Ed.* **63**, e202410835 (2024).
46. Searles, K. *et al.*, Highly Productive Propane Dehydrogenation Catalyst Using Silica-Supported Ga–Pt Nanoparticles Generated from Single-Sites. *J. Am. Chem. Soc.* **140**, 11674-11679 (2018).
47. Nakaya, Y. *et al.*, High-Entropy Intermetallics Serve Ultrastable Single-Atom Pt for Propane Dehydrogenation. *J. Am. Chem. Soc.* **144**, 15944-15953 (2022).
48. Rochlitz, L. *et al.*, A Robust and Efficient Propane Dehydrogenation Catalyst from Unexpectedly Segregated Pt_2Mn Nanoparticles. *J. Am. Chem. Soc.* **144**, 13384-13393 (2022).
49. Liu, L. *et al.*, Regioselective generation and reactivity control of subnanometric platinum clusters in zeolites for high-temperature catalysis. *Nat. Mater.* **18**, 866-873 (2019).
50. Sun, Q. *et al.*, Subnanometer Bimetallic Platinum–Zinc Clusters in Zeolites for Propane Dehydrogenation. *Angew. Chem. Int. Ed.* **59**, 19450-19459 (2020).
51. Wang, Y. S., Z. P. Hu, X. W. Lv, L. Chen, Z. Y. Yuan, Ultrasmall PtZn bimetallic nanoclusters encapsulated in silicalite-1 zeolite with superior performance for propane dehydrogenation. *J. Catal.* **385**, 61-69 (2020).
52. Fan, X. Q. *et al.*, Mn-doping induced changes in Pt dispersion and Pt_xMn_y alloying extent on Pt/Mn-DMSN catalyst with enhanced propane dehydrogenation stability. *J. Catal.* **389**, 450-460 (2020).
53. Zeng, L. *et al.*, Stable anchoring of single rhodium atoms by indium in zeolite alkane dehydrogenation catalysts. *Science* **383**, 998-1004 (2024).
