# Supplementary material for: Space-confined synthesis of sinter-resistant high-entropy nanoparticle library
Source: Nat Commun. 2025 Aug 11;16:7383. doi: 10.1038/s41467-025-62729-3 (PMC12339747; doi:10.1038/s41467-025-62729-3)
Supplement: Supplementary file 2 — Description of Additional Supplementary Files [file 41467_2025_62729_MOESM2_ESM.pdf]

Description of Additional Supplementary Files

File Name: Supplementary Movie 1
Description: Spontaneous droplet growth on an open non-wetting surface, simulated using the fluid–solid lattice Boltzmann (FSLB) method.

File Name: Supplementary Movie 2
Description: Confined droplet growth within mesoporous channels, simulated using the FSLB method.

File Name: Supplementary Movie 3
Description: Stable confinement of droplets comparable in size to the pore diameter, simulated using the FSLB method.

File Name: Supplementary Movie 4
Description: 3D reconstruction by electron tomography showing that high-entropy nanoparticles are confined within the mesopores of MCM-41 in the Pt-Quinary-HEOs@MCM41 sample

File Name: Supplementary Movie 5
Description: 3D reconstruction by electron tomography showing that high-entropy nanoparticles are confined within the ZSM-5 crystal in the Pt-Senary-HEOs@ZSM-5 sample.

File Name: Supplementary Movie 6
Description: Liquid metal droplets evolve into 1D nanocolumns in non-wetting nanopores as precursor loading increases, simulated using the FSLB method.

File Name: Supplementary Movie 7
Description: 3D reconstruction by electron tomography confirming the confinement of high-entropy nanoparticles within the mesopores of MCM-41 in the Pt-QuinaryHEOs@MCM-41-spent.
